# Modeling and analysis of Hi-C data by HiSIF identifies characteristic promoter-distal loops

**DOI:** 10.1186/s13073-020-00769-8

**Published:** 2020-08-12

**Authors:** Yufan Zhou, Xiaolong Cheng, Yini Yang, Tian Li, Jingwei Li, Tim H.-M. Huang, Junbai Wang, Shili Lin, Victor X. Jin

**Affiliations:** 1 Department of Molecular Medicine, University of Texas Health San Antonio, San Antonio, TX 78229 USA; 2 Department of Pathology, Oslo University Hospital – Norwegian Radium Hospital, Montebello, 0310 Oslo, Norway; 3 Department of Statistics, The Ohio State University, Columbus, OH 43210 USA

## Abstract

Current computational methods on Hi-C analysis focused on identifying Mb-size domains often failed to unveil the underlying functional and mechanistic relationship of chromatin structure and gene regulation. We developed a novel computational method HiSIF to identify genome-wide interacting loci. We illustrated HiSIF outperformed other tools for identifying chromatin loops. We applied it to Hi-C data in breast cancer cells and identified 21 genes with gained loops showing worse relapse-free survival in endocrine-treated patients, suggesting the genes with enhanced loops can be used for prognostic signatures for measuring the outcome of the endocrine treatment. HiSIF is available at https://github.com/yufanzhouonline/HiSIF.

## Background

Chromosome conformation capture (3C)-based genome-wide technologies, including Hi-C or TCC [1–5], ChIA-PET [6, 7], HiCap [8], Capture-C [9, 10], and 3C-seq methods [11], have greatly expanded our understanding of the basic principles of three-dimensional (3D) genome organization, providing new insights into how chromosomes fold within distinct territories [1, 3, 12]. Studies further revealed chromosome territories are distributed over spatial compartments or partitioned into topological associated domains (TADs) [2, 5]. However, such a large domain usually embedded with multiple genes is hard to associate chromosomal interactions with transcriptional control at the individual gene level. Although a recent study used an in situ Hi-C protocol to achieve 1–5 kb resolution of genomic interaction [5], such protocol requires an extremely high sequencing depth of ~ 5 billion paired-end reads for each sample, making it impractical for many studies. Computational and statistical modeling on relatively low sequence depth data showed that Hi-C data are able to identify interacting genomic regions at a resolution of 10–20 kb [4, 5, 13]. To achieve such a high resolution, three major challenges are posed for any computational and statistical modeling. The first is to filter out background ligations and biases [14–16]. The second one is to remove random ligation interactions from proximity-based ligations since they artificially add a false count rate for the true interactions. The last and most crucial one is to quantify the significant chromosomal interactions. Methods include analyzing high-resolution contact frequency map for significant pixel counts via graphical processing unit enabled image analyzing algorithms [5], or searching for pairs of regions that have more Hi-C reads between them than would be expected by a background model [15]. Typically, statistical models are dependent on the sequencing depth used to prepare the Hi-C Library. However, it is imperative to have better probabilistic models in order to identify both statistically and biologically significant interactions.

One big advantage of identifying unbiased chromatin interactions at a higher resolution is to allow us to associate each pair of chromatin interaction fragments with individual gene looping, a transcription paradigm achieved by the combinatorial interactions of DNA-binding transcription factors (TFs) bound to distal regions with other TFs bound to proximal regions [17–19]. Indeed, several studies have demonstrated that cell type-specific gene expression processes may be intricately related to the 3D organization of the genome [20–28]. However, many of these large-scale structural studies were limited on domain-based analysis and thus failed to unveil the underlying functional and mechanistic relationship of higher order chromatin structure and specific gene regulation. We recognized that some anchored-specific 3C techniques such as ChIA-PET [6, 7], 5C [29], HiCap [8], or Capture-C [9, 10] can partially address the above concerns. For example, a recent study found that super enhancer-driven genes generally occur within chromosomal domains formed by the looping of genomic regions that are bound by CTCF and cohesion by using ChIA-PET [7]. However, one critical question remains to be answered, i.e., does there exist such non-promoter-centered chromatin interactions or distal-distal loops, if yes, do they have any biological meaning? Therefore, it is imperative to develop novel computational approaches to identify distinct classes of chromatin interactions from all-all interactions in Hi-C/TCC data.

Here, we develop a computational model, Hi-C Significant Interacting Fragment (HiSIF), on Hi-C data analysis, including a Poisson Mixture Model (PMM) [30, 31], with an Expectation Maximization (EM) algorithm [32, 33] followed by a power-law decay background model [34] to filter out background-ligation events. We test and evaluate HiSIF on publically available Hi-C [35] and in situ Hi-C [5] data and compare its performance to some existing programs. We then apply it on newly generated in situ Hi-C data in breast cancer tamoxifen-sensitive and resistant cells.

## Methods

### Pre-processing Hi-C data

Four publicly available human Hi-C datasets representing different experimental protocols and sequencing depths were downloaded, including Hi-C data in MCF10A and MCF7 cells [36], hESC cells [35], and in situ Hi-C data in GM12878 cells and K562 cells [5]. Raw and processed Hi-C data for MCF7 and MCF7-TamR cells is deposited in GEO under accession number GSE108787 [37]. All Hi-C data were aligned to human genome hg19 and pre-processed using the hiclib pipeline [16], and formatted as an appropriate input to HiSIF. We kept high-quality PE reads with a criterion of MAPQ > 30 during the iterative mapping process. A summary of datasets was listed in the Additional file 1: Table S1.

### Plotting the distribution of Hi-C data

Ultrasonic Fragments (USFs) are defined as those uniquely mapped paired-end reads located within the closest restriction enzyme digestion sites. The USF counts are the sum of the mapped reads within USFs, and the frequency of USF counts was plotted as distribution of USF counts frequency. The genomic distance between two end reads of the pair was calculated. The ratio of the counts of each genomic distance to the total counts of all genomic distances was computed as ligation probability. The ligation probability in various genomic distances was plotted as a distribution of genomic distance.

### Generating Hi-C subsets in specific sequencing depth

Public datasets represent limited cases of sequencing depths. To evaluate the performance, HiSIF were tested with different sequencing depths. The Reservoir Sampling algorithm was used to randomly extract PE reads from Hi-C data for generating subsets in specific sequencing depth [38]. To minimize the effect of uneven sequencing depth of the subsets, each of subsets contains 10 samples with the same sequencing depth. Here, we simply define the sequencing depth as linearly proportional to the number of PE reads.

### Developing a PMM with a power-law decay background

We developed a PMM combined with a power-law decay background to define chromatin interactions for Hi-C data. In this model, the proximate ligation events and random ligation events are considered as two independent Poisson distributions and thus the overall ligation events could be represented by a latent class mixture model with two hidden variables. Here we define a proximate ligation as a ligation between two ends that are spatially adjacent to each other and a random ligation as a ligation between two randomly interacting DNA fragments. The EM algorithm was used to estimate the proportion and the parameters of the two independent Poisson distributions.

We considered each valid USF as an independent observation *d*_*l*_ (score for the *l*^th^ interaction of *N* number of data points), *ω*_*k*_ determines which component of the mixture is *y*_*j*_ originated (weight component), *k* represent the *k*^*th*^ component of the mixture model with *k* number of mixtures. In HiSIF, *k* = 1,2 for random and proximate ligation events. Using the sum of probability *ω*_*k*_ can be written as

1 $$ {\sum}_{\mathrm{k}=1}^2{\omega}_k=1 $$

The likelihood function for a two-component Poisson mixture model can be written as

2 $$ L\left(D:\theta \right)={\prod}_{\mathrm{l}=1}^N{\sum}_{\mathrm{k}=1}^2{\omega}_k{g}_k\left({D}_l:{\lambda}_k\right) $$

where *g*_*k*_(*D*_*l*_ : *λ*_*k*_) is the probability density function of the Poisson distribution with mean *λ*_*k*_ and *D*_*l*_ is the set of all interaction scores. By maximizing the above likelihood and assuming initial mixture parameters and mixing proportions, the following recursive formulas could be derived to update the parameters for the next iteration:

3 $$ {\omega}_k=\frac{1}{N}{\sum}_{\mathrm{l}=1}^Np\left(k\vee l\right) $$

4 $$ {\lambda}_k=\frac{\sum_{\mathrm{l}=1}^Np\left(k\vee l\right){D}_l}{\sum_{\mathrm{l}=1}^Np\left(k\vee l\right)} $$

where *p*(*k* ∨ *l*) denotes the conditional probability of selecting component *k* given the observation *D*_*l*_. We used Bayesian Information Criterion (BIC) to determine the number of components (target sites) within a score enriched region. A detailed mathematical derivation of the EM algorithm is explained in Supplementary Methods. It is compulsory to achieve maximum likelihood in certain iteration steps. We used this parameter to measure the performance of the HiSIF algorithm.

We can also control the ratio of false-positive interactions by false discovery rate (FDR) statistic. There are two principal FDR statistic: global FDR and local FDR. The global FDR, proposed by Benjamini and Hochberg, consists of a procedure that controls the global proportion of false-positive findings based on the *p* value rank. The local FDR, a conceptually different approach, is based on estimating the probability density function of the FDR directly from the actual data. Since there is no biological replication, no population inference can be made and hence it is invalid for us to calculate the *p* value [39]. Therefore, the local FDR method is used in the HiSIF algorithm. For a given USF score, we can define its FDR as:

5 $$ {\mathrm{FDR}}_d=\frac{{\mathrm{FP}}_d}{{\mathrm{FP}}_d+{\mathrm{TP}}_d} $$

where FP_*d*_ is the probability density function of false-positive FP at USF score *d* and TP_*d*_ is the probability density function of true-positive TP. The fragment threshold rate (FTR) is the count number threshold for every fragment. If count number in a specific fragment is far less than the FTR, the interaction will be considered as an amplification of noise. If the count number is close to but less than the FTR, the interaction will be considered as a weak interaction. Only when the count number is larger than the FTR, the fragment will be treated as a significant interaction fragment candidate. There may be some false-positive events in the candidates. Therefore, the FDR is designed to remove the false-positive fragments from the significant interaction fragment candidates.

Once determining the appropriate mixture parameters, we can estimate the probability density function of significant fragment scores and treat that as the true-positive TP. To obtain the distribution of the false-positive FP, HiSIF generates data sets by randomly extracting one of the fragment scores. Repeating the process a large number of times (*Np*), a set of test statistics is obtained, whose probability density function defines the empirical distribution of the null hypothesis or false-positive.

Then we can eliminate random ligation events for a user-specified FDR threshold. The likelihood function *L* of two restriction fragments *F*_*i*_ and *F*_*j*_ forming an interaction can be written as follows:

6 $$ L\left({F}_i:{F}_j\right)={\prod}_{F_k\in F}^K{\left(1-E\right)}^{k-j}P\left({F}_i,{\mathrm{F}}_j\right) $$

where *F*_*i*_, *F*_*j*_, *F*_*k*_, are the *i*^th^, *j*^th^, *k*^th^ digested restriction fragments, *F* represents a set of digested fragments surrounding *F*_*j*_, *E* is the digestion efficiency of the restriction fragments, and *P*(*F*_*i*_, F_*j*_) is the probability of ligation between *F*_*i*_ and *F*_*j*_ determined by the power-law distribution. This will allow us to remove further background ligation events beyond a threshold of likelihood. A more detailed procedure is described in Supplementary Methods. The source codes for HiSIF can be accessed from https://github.com/yufanzhouonline/HiSIF [40].

### Defining the HiSIF resolution

The resolution of a Hi-C dataset highly depends on the protocol used (Hi-C/TCC/in situ) and was poorly defined in the literature thus far. In most Hi-C data analysis, restriction fragments were aggregated into a fixed bin size defined as the resolution [41]. So the number of reads corresponding to a particular bin size determines the quality or the resolution of a Hi-C dataset. If the number of unique Hi-C molecules in the sample is high, extra sequencing will add more quality and a very high resolution can be achieved. On the other hand, if the total number of unique Hi-C molecules in the sample is low, a good resolution cannot be achieved by high sequencing depth. Moreover, proximity-based Hi-C molecules represent only a fraction of any Hi-C library and are usually masked by molecules formed by random ligations. Thus, depending on the protocol and particular background filtering methods, two sequenced libraries with the same number of reads may contain a different number of informative interactions. If randomly ligated molecules present in large numbers, they may completely mask true interactions. Since HiSIF removes most of the random ligations, highly specific informative interactions can be preserved for the final data analysis. Unlike defining a binned Hi-C interaction matrix, HiSIF uses a range of resolutions and the optimal resolution can be seen as a local maximum of the constructed resolution range. To demonstrate the construction of resolutions (Additional file 1: Fig. S1-right) we illustrated a one-dimensional view of a segment of Hi-C interactions with eight restriction fragments, F1–F8, with the respective lengths, L1–L8. Particular USFs corresponding to F1–F8 were shown in different colors above and below restriction fragments (blue for F1–F8, green F2–F7, red F3-F5, and purple F4–F6). If the corresponding score related to the cut-off FDR threshold is 3, all the interactions higher than score 3 is retained as proximate ligation events and less than 3 is thrown out as random ligation events. In this example illustration, F4–F6 is a random ligation event (score = 3) and F1–F8 (score = 4), F2–F7 (score = 4), F3–F5 (score = 5) are proximate ligation events with fragment threshold rate (FTR) is equal to 3. Resolution for the F3–F5 interaction is L3, L5 for side 1 and 2, respectively. If consecutive fragments have the same score we merge them (similar to peak summit in ChIP-seq) together into one interaction. Thus the resolution of the other interaction is L1 + L2, L7 + L8 for side 1 and 2, respectively.

### Receiver operating characteristic (ROC) curve and the area under the curve (AUC)

The ROC curve analysis for the methods of HiSIF and HICCUPS (Module of Juicer Tools Version: 1.13.02) and Fit-Hi-C (Version: 2.0.7) [42] was performed on the K562 Hi-C data [5], ENCODE K562 Histone, and ChIA-PET data [14] with the CTCF ChIA-PET loops as the reference. The putative loops were defined as interactions of the anchor labeled by the promoter histone marker H3K4me3 within +/− 5 kb of CTCF peak summit and the enhancer labeled by histone marks H3K27ac/H3K4me1 within +/−100 kb of CTCF peak summit. The positive loops were defined as the overlapping loops with CTCF ChIA-PET. According to the overlapping or not, all putative loops could be classified as follows: true-positive (TP)—the methods and ChIA-PET both identified; true-negative (TN)—the methods and ChIA-PET both non-identified; false-positive (FP)—the methods identified but ChIA-PET not; and false-negative (FN)—the ChIA-PET identified but the methods not. TPR is the ratio of TP to the sum of TP and FN. FPR is the ratio of FP to the sum of FP and TN. AUC score is the area of ROC curve.

### Chromosome conformation capture coupled with quantitative PCR (3C-qPCR)

3C-qPCR experiments were referred to chromosome conformation capture assay as previously described [37, 43]. Briefly, ten million cells were collected and then fixed with 1% formaldehyde. Cells were lysed with 0.2% Igepal CA630 to get the pelleted nuclei followed by solubilization with 0.3% sodium dodecyl sulfate (SDS). The solubilized nuclei were diluted with 2% Triton X-100 and then digested with 400 U HindIII. After diluting again, the genomic DNA were ligated with T4 DNA ligase. The ligated DNA was de-crosslinked and purified followed by dissolving in 10 mM Tris-HCl to get 3C DNA libraries. These libraries were used as the templates for the subsequent quantitative PCR. The primers involved in the 3C-qPCR experiments were listed in the Additional file 1: Table S2.

### Reverse transcription quantitative PCR (RT-qPCR)

The total RNA was extracted from ten million MCF7 or MCF7-TamR cells with Quick-RNA MiniPrep kit (Zymo Research, # R1054). The extracted RNA was then used as templates for qPCR performed with SuperScript III Platinum SYBR Green One-Step qRT-PCR Kit (Invitrogen, # 11736-059). The primers involved in the RT-qPCR experiments were listed in Additional file 1: Table S3.

## Results

### Overview of HiSIF algorithm

**Fig. 1:**
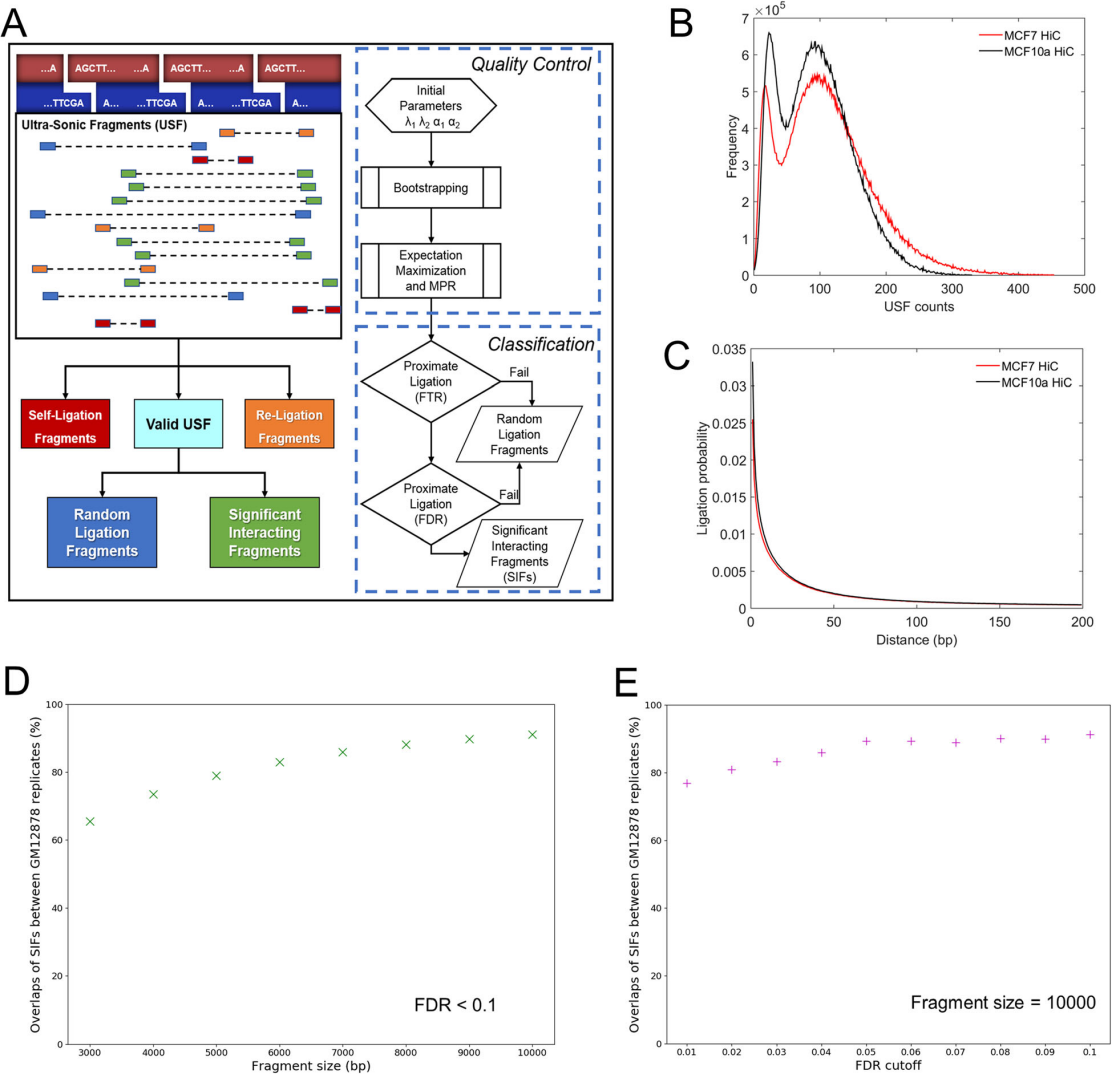
An overview of the HiSIF algorithm. **a** Left: the schematic diagram of HiSIF. Right: the flowchart of the HiSIF algorithm including two modules: Quality Control and Classification of Hi-C interactions. **b** A distribution of USF counts frequency where a clear distinction of two Poisson means was observed in both MCF7 and MCF10a datasets. **c** Distribution of the genomic distance between every pair of ligated DNA segments where the result showed that the probability of background ligations follows a power law distribution. **d** The percentage of overlaps of SIFs between GM12878 replicate 1 and replicate 3–9 in various fragment sizes. **e** The percentage of overlaps of SIFs between GM12878 replicate 1 and replicate 3–9 in various FDR cutoffs

We developed a novel computational and statistic algorithm for processing Hi-C data, HiSIF, composed of two main modules (Fig. 1a), Quality Control and Classification of Hi-C interactions. HiSIF did not include the mapping of initial FASTQ files due to various publicly available genomic mapping tools. The Quality Control module initializes the parameters for the PMM, optimizes them based on the individual dataset, and characterizes different Hi-C interactions into self-ligation, re-ligation, and valid-ligation events. In the Classification module, valid-ligation events are further quantified as random-ligation and proximate-ligation interactions using a PMM and a power-law decay background model. Random ligations are eliminated based on a fragment threshold rate (FTR). Significant Interacting Fragments (SIFs) are then identified from proximate-ligations with a false discovery rate (FDR) which can be defined by users.

#### Detection of significant interactions

After the Quality Control of Hi-C sequencing fragments (Supplementary Methods, and Additional file 1: Fig. S1–2), i.e., any paired-end (PE) reads uniquely mapped to two particular restriction fragments as Ultrasonic Fragments (USFs), a score (USF counts) was assigned to a valid Hi-C interaction (Fig. 1b). We observed a clear distinction of two Poisson means being visible in the frequency distribution plot. However, the genomic distance between every pair of ligated DNA segments, i.e., background ligations, follows a power law distribution (Fig. 1c), consistent with the initial Hi-C study [1]. This distribution also shows a high background ligation rate between closely positioned restriction fragments. Thus, we constructed a power law decay background model to further filter out background ligations that are formed due to linear closeness.

FTR and FDR are two inter-dependent measures used for classifying highly specific proximate ligation events from random ligation events. To determine the values of FTR and FDR, we apply the EM algorithm to extract the mean scores for random and proximate ligation events. HiSIF pre-sets the initial Poisson means for random and proximate ligations, then uses a bootstrap-like scheme to make the measures more robust. Suppose that we had *M* data points (*M* USFs), we randomly picked one of the data points and recorded it, then repeated this process by *M* times and got *M* recorded data points. These *M* points consisted of one re-sampling set of the original dataset. Now, we applied the EM to this new data sample and measured the corresponding parameters. After this, we started the second round, getting another re-sampling set with *M* data points in it and measuring the parameters using PMM again. We repeated this process by *N* times resulting in *N* estimates of each parameter. We discarded those outliers’ estimates (estimates beyond the upper and lower inner fences) for each parameter and used the mean of good estimates as the value of each parameter. Using this scheme, the resulted parameter estimates were more optimized and robust at a cost of a tolerable increase in computation time.

#### The overlapping of biological replicates

The overlapping of biological replicates could be used to identify the reproducibility of the algorithm. We performed the overlapping analysis for the SIFs of the replicate 1 and the replicate 3–9 of GM12878 (Additional file 1: Fig. S3, Additional files 2 and 3). The overlaps of SIFs increased from 65 to 91% with the extension of fragment size from 3 K to 10 K at FDR < 0.1 (Fig. 1d). More than 80% overlaps of SIFs were obtained when FDR cutoff is between 0.02 and 0.1 with the fragment size is 10 K (Fig. 1e). These results suggest that HiSIF is highly reproducible for the identification of significant interaction fragments.

#### Performance of FTR and FDR

**Fig. 2:**
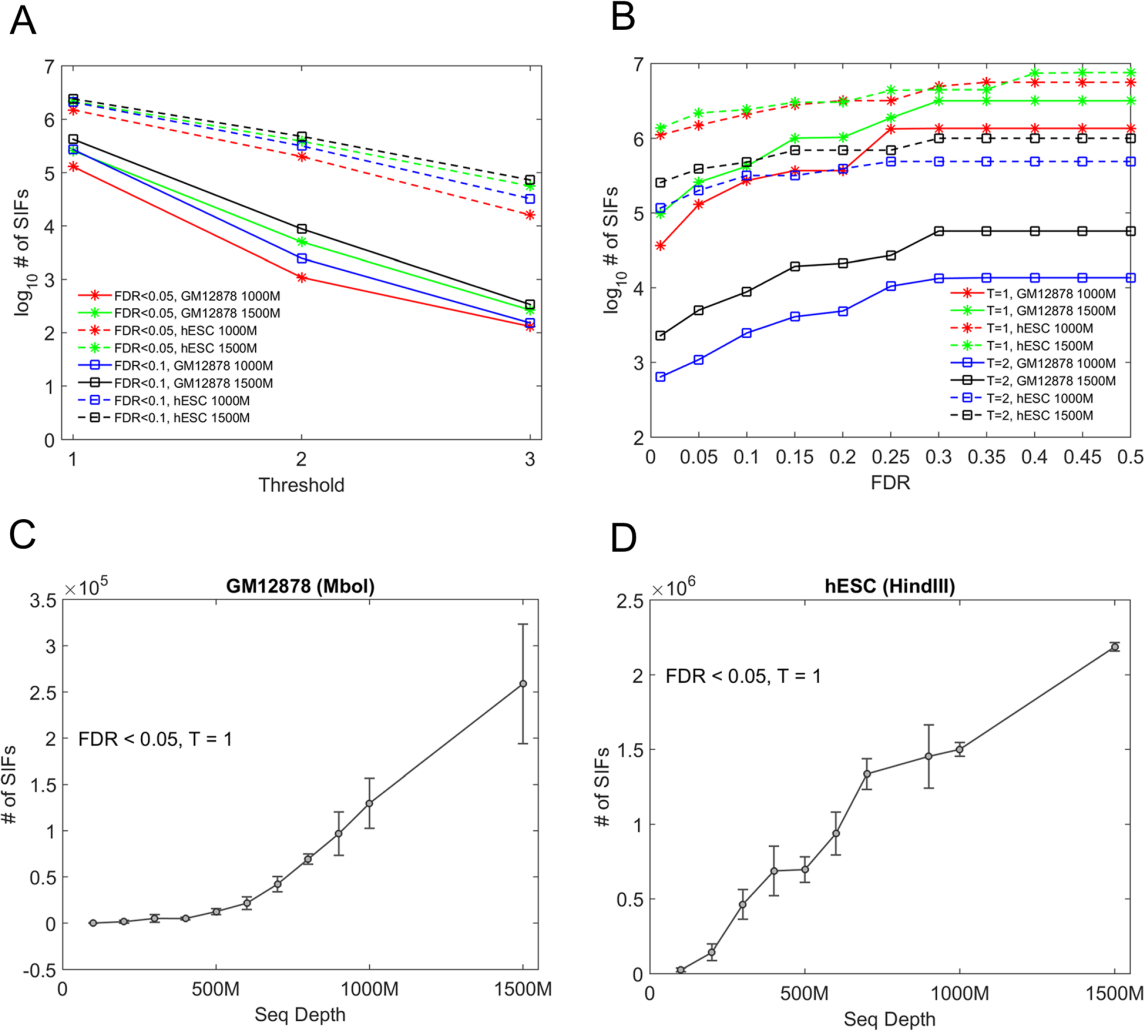
Performance of FTR and FDR of HiSIF. **a** The number of SIFs at various FTR thresholds. The number of SIFs decreased rapidly along with increasing FTR threshold. **b** The number of SIFs at various FDRs. It was clear that most SIFs detected by HiSIF were at FDR less than 0.25. **c**, **d** The number of SIFs with various sequence depths in GM12878 and hESC cells. The number of SIFs increased rapidly along with increasing sequence depth. However, for a given sequence depth, SIFs detected in hESC Hi-C data (HindIII) were nearly an order of magnitude higher than those detected in GM12878 in situ Hi-C data (MboI)

We further evaluated the performance of the FTR and FDR by testing different Hi-C protocols (Hi-C with HindIII restriction enzyme vs in situ Hi-C with MboI/DpnII restriction enzyme) and sequencing depths. For a given FDR regardless of Hi-C protocols and sequence depths, we found the number of SIFs rapidly decreases with an increase of FTR and the log10 number of SIFs almost linearly decreases (Fig. 2a), suggesting FTR is a proper parameter for defining the significance level. However, for a given FDR and a given sequence depth, we detected much more SIFs for hESC Hi-C data (HindIII) [35] than for GM12878 in situ Hi-C data (MboI) [5], illustrating that in situ Hi-C with MboI lacks an advantage of detecting more SIFs despite of its higher sequencing depth. For a given FTR, the number of SIFs gradually increases with an increase of FDR (Fig. 2b) but towards steady at a FDR of 0.25 for both Hi-C protocols, demonstrating that the performance of HiSIF is reliable and effective. We also found that the number of SIFs increases rapidly along with an increase of sequence depth (Fig. 2c, d). However, for a given sequence depth, we detected nearly ten times SIFs for hESC Hi-C data than for GM12878 in situ Hi-C data. One possible reason is that MboI/DpnII data have much more random ligation events which would hinder the correct estimation of the significant interactions in the mixture model.

### Comparison with other available Hi-C tools

The performance of HiSIF was compared with two publicly available software tools, HiCCUPS [5] and Fit-Hi-C [13]. HiCCUPS assumes a significant interaction as a peak at two-dimensional contact matrices and computes its enrichment score comparing to its neighboring regions. In this sense, HiCCUPS is very stringent and usually detects very small number of interactions. Fit-Hi-C assigns statistical confidence estimates to mid-range intra-chromosomal contacts by jointly modeling the random polymer looping effect and previously observed technical biases.

**Fig. 3:**
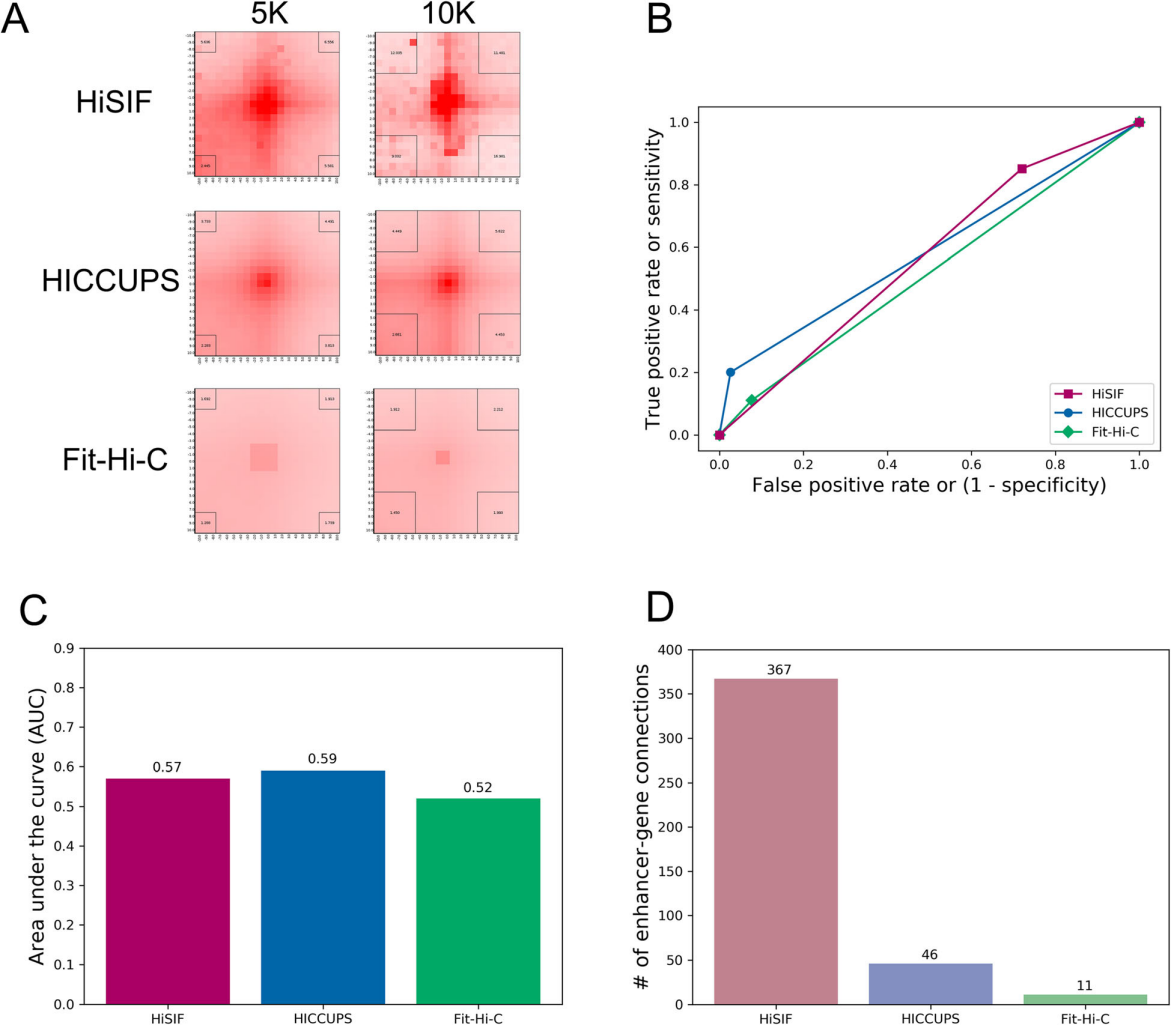
A comparison of performance of HiSIF with other available Hi-C tools. **a** Aggregate peak analysis (APA) of three tools. Four numbers in the corners are the ratio of the central pixel to the pixels in the square of corner. The number of lower left corner is defined as the APA value. APA was based on the ENCODE K562 data. **b** Receiver operating characteristic (ROC) curve of three tools. **c** The area under the curve (AUC) of ROC curve. **d** The number of enhancer-gene connections of CRISPRi-FlowFISH in three tools

For K562 in situ Hi-C data with sequencing depth of 1.4 billion PE reads (Additional file 1: Table S1) [5], 6473 SIFs were identified by HiCCUPS, 172,084 by Fit-Hi-C at resolution 20 K and *q* value < 1 × 10^− 14^ and 176,078 by HiSIF at FDR < 0.001 and FTR 1 (Additional files 4, 5 and 6). We performed an aggregate peak analysis (APA) of Hi-C loops on the K562 data, and found that HiSIF, HICCUPS, and Fit-Hi-C have the APA value of 2.445, 2.283, and 1.288 in 5 K resolution and 9.032, 2.661, amd 1.450 in 10 K resolution, respectively (Fig. 3a), demonstrating that HiSIF has the most enriched focal point of chromatin loops.

Receiver operating characteristic (ROC) curve has been widely used for the analysis of sensitivity and specificity in evaluating the accuracy of an algorithm. The curve plots the true positive rate (TPR) or sensitivity versus the false positive rate (FPR) or 1—specificity. We performed the ROC curve analysis of HiSIF, HICCUPS, and Fit-Hi-C on the ENCODE K562 data [14] with the CTCF ChIA-PET loops as the reference (Fig. 3b). Clearly, HiSIF has the highest sensitivity than HICCUPS and Fit-Hi-C. We further performed the area under the curve (AUC) of ROC curve which is the probability of the classifier model. HiSIF has the approximate value as HICCUPS but obviously higher value than Fit-Hi-C (Fig. 3C) indicating HiSIF is a better tool for the looping identification.

CRISPRi-FlowFISH protocol could be used to identify functional enhancer and gene/promoter connections [44]. We used it as a standard to evaluate the quality of the identified loops by all three tools. HiSIF covered 367 enhancer-gene connections identified by CRISPRi-FlowFISH, but HICCUPS and Fit-Hi-C only covered 46 and 11, respectively (Fig. 3d), suggesting that HiSIF identified more biological meaningful loops than HICCUPS and Fit-Hi-C did.

We further investigated the enhancer-promoter interactions, promoter-promoter interactions, enhancer-enhancer interactions, and convergent CTCF motifs among these method-shared and method-specific interactions for GM12878 and hESC. HiSIF identified more enhancer-promoter interactions than Fit-Hi-C in both GM12878 and hESC (Additional file 1: Figs. S4–5, Columns 6 and 8), HiSIF identified more enhancer-promoter interactions than HICCUPS in GM12878 but not in hESC (Additional file 1: Figs. S4–5, Columns 2 and 4). Method-shared interactions have more convergent CTCF motifs than method-specific interactions (Additional file 1: Fig. S6). These results suggest that HiSIF could be used for the identification of putative enhancer-promoter loops effectively. In addition, HiSIF has less percentage of loops within TAD (topologically associating domain) boundaries than HICCUPS, and Fit-Hi-C as well (Additional file 1: Fig. S7).

### The application of HiSIF to in situ Hi-C data in ERα + breast cancer cells

**Fig. 4:**
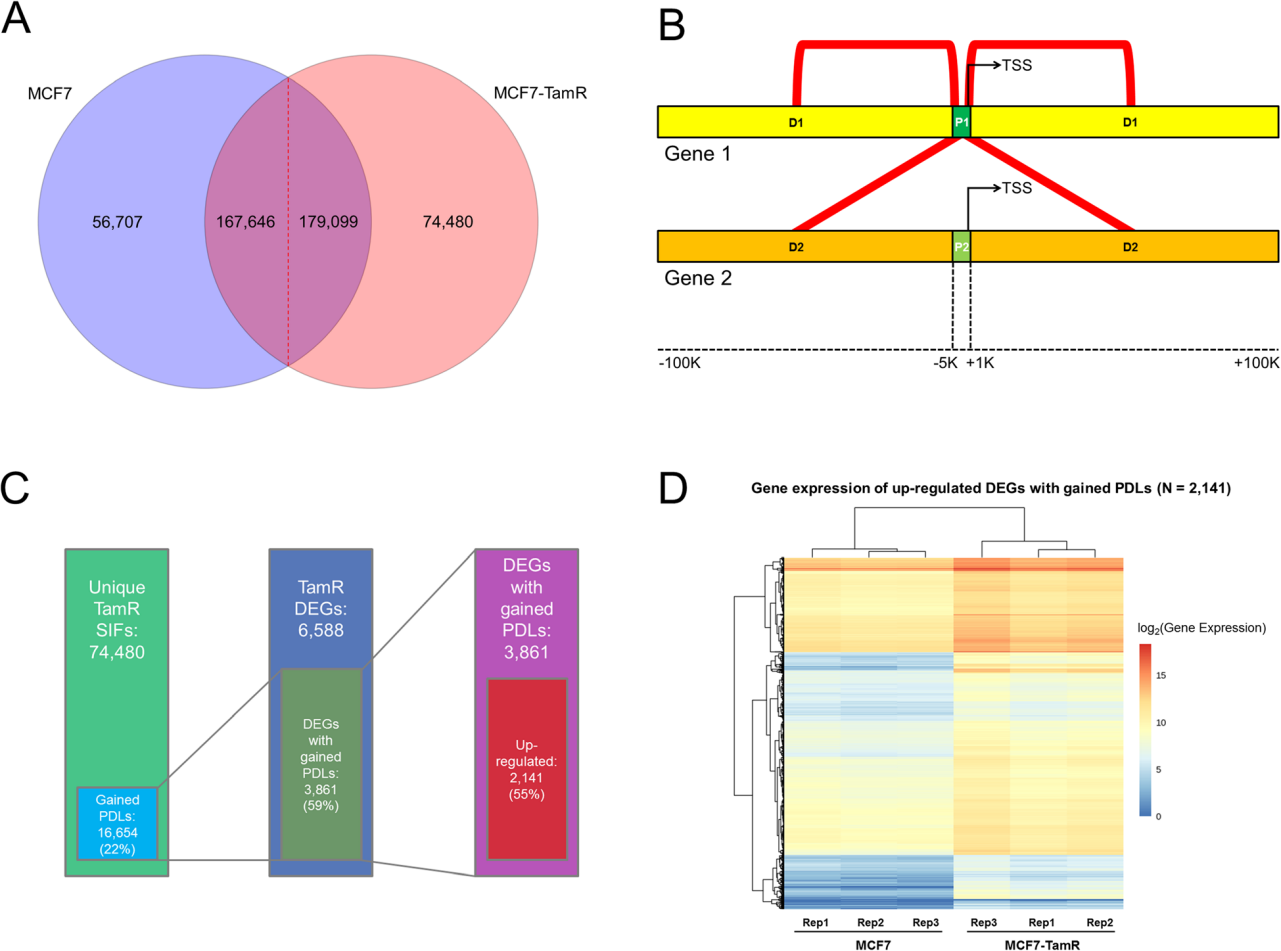
The application of HiSIF in Hi-C data in ERα + breast cancer sensitive cells MCF7 and resistant cells MCF7-TamR. **a** The Venn diagram of SIFs of MCF7 and MCF7-TamR. There are two numbers in the regions separated with red dash lines because the overlapped regions have different numbers of SIFs in various cells. **b** The diagram representing two categories of promoter-distal loops (PDLs) of the promoter-containing SIFs according to their distance relative to the TSS of the promoter and whether the non-promoter end is closest to the same gene (P1D1 or P1D2). The promoter region (P1/P2) was defined as − 5 K/+ 1 K to TSS. The distal region (D1/D2) was defined as +/− 100 K to TSS excluding the promoter region. PDLs were indicated with red lines. **c** The number of gained PDLs and differentially expressed genes (DEGs) with gained PDLs in MCF7-TamR cells. **d** The heatmap of gene expression of upregulated DEGs with gained PDLs

We have applied our HiSIF in ERα + breast cancer cells MCF7 and their tamoxifen-resistant cells MCF7-TamR [37] (Additional file 1: Fig. S8–9). We applied HiSIF to these data with the optimal parameters (FTR = 1 and FDR < 0.1) and detected a total of 224,353 SIFs for MCF7 and 253,579 for MCF7-TamR, respectively. Common and unique SIFs of these two cells were also identified with 56,707 (25%) MCF7-unique and 74,480 (29%) MCF7-TamR-unique (Fig. 4a).

We further examined the promoter-centric SIFs or promoter-distal loops (PDLs), whereas they are defined in the following: one end of SIF is within a promoter (defined as − 5 K/+ 1 K to transcription start site (TSS)) and the other end is within a non-promoter region (defined as +/− 100 K to TSS). Thus, the PDLs are further classified into two types, P1D1 and P1D2. If the non-promoter end is closest to the same gene, this PDL is defined as P1D1; if the non-promoter end is closest to the other gene, this PDL is defined as P1D2 (Fig. 4b). Interestingly, we found 16,654 gained PDLs (22% of MCF7-TamR unique SIFs) and 3861 differentially expressed genes (DEGs) associated with gained PDLs in MCF7-TamR cells (Fig. 4c, Additional file 7). More than 55% DEGs with gained PDLs were upregulated (Fig. 4d, Additional file 8) in MCF7-TamR cells, indicating that these gained loops might functionally contribute to the process of acquired tamoxifen resistance.

### The characteristics of the genes associated with gained loops

**Fig. 5:**
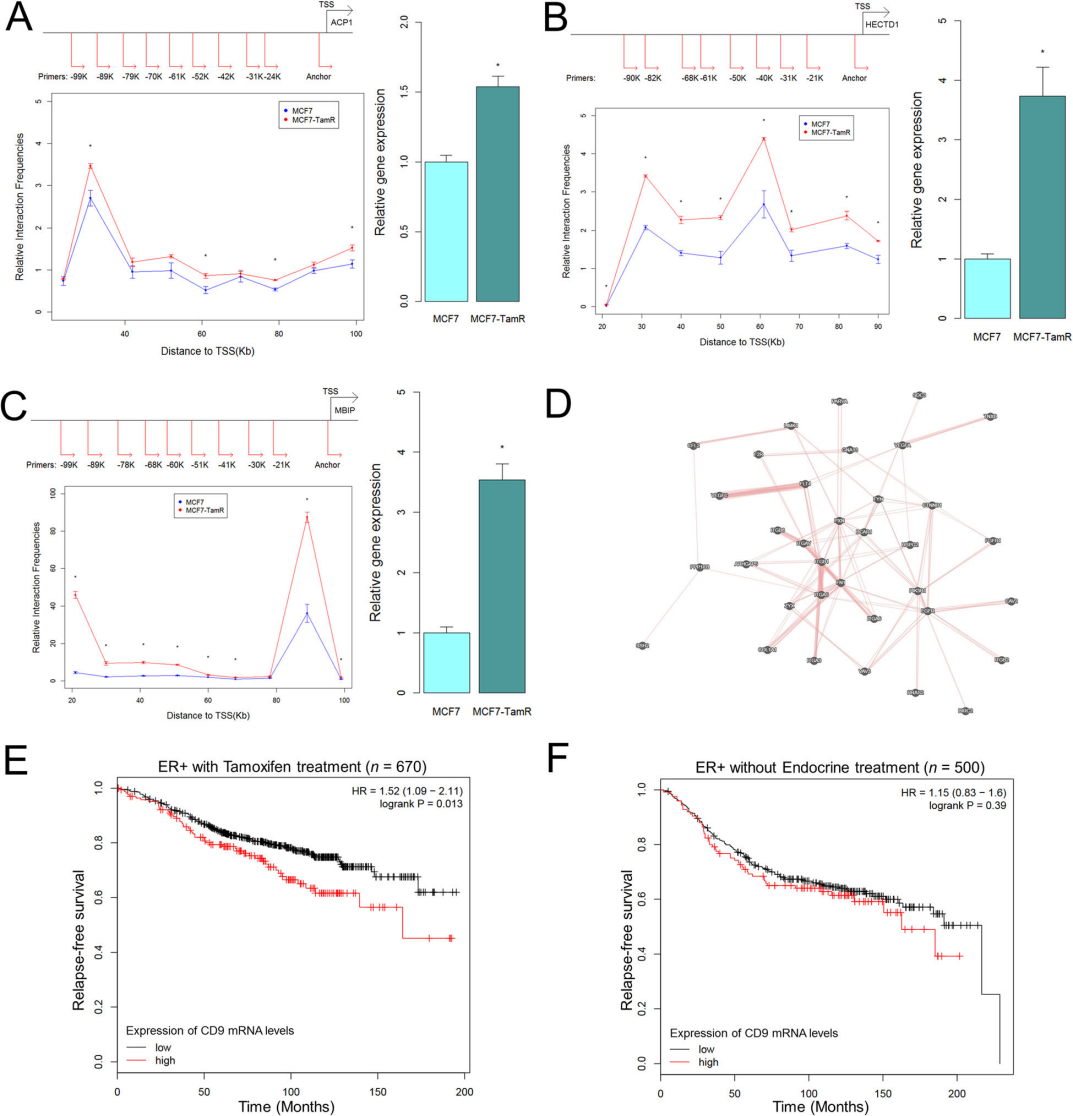
The genes with gained PDLs in MCF7-TamR cells. **a**-**c** Experimental validation on the selected gained looping events in MCF7-TamR cells by 3C-qPCR and RT-qPCR. Left top panel: illustration of primers applied in 3C-qPCR experiments. Left bottom panel: 3C-qPCR. Right panel: RT-qPCR. TSS: transcription start site. GAPDH was used as the control for both relative interaction frequencies and relative gene expression. *p < 0.05 (two-sided *t* test). Error bars represent standard error of the mean (S.E.M.) with three experiments. Detected genes: **a** ACP1, **b** HECTD1, **c** MBIP. **d** Signaling pathway networks of the core components of three signaling pathways: ECM receptor interaction, regulation of actin cytoskeleton and focal adhesion enriched by GSEA with the upregulated genes of gained PLDs in MCF7-TamR cells. **e** Relapse-free survival analysis of CD9 mRNA levels in ERα + patients receiving only Tamoxifen but without chemotherapy. The patients (*n* = 670) were stratified by mRNA levels at the top quartile (25%) vs. the rest (75%). *p* value was determined by the log-rank test. Analysis was referred to the published paper [34]. HR, hazard ratio. **f** Same analysis of CD9 mRNA levels in ERα + patients without endocrine treatment (*n* = 500)

We further performed the experimental validations on three selected genes with gained promoter-distal loops within the 100 kb upstream of TSS, ACP1, HECTD1, and MBIP, in MCF7-TamR vs MCF7 cells by 3C-qPCR and RT-qPCR (Fig. 5a–c). Clearly, these gained loops were confirmed and showed higher interaction frequencies in MCF7-TamR cells than in MCF7 cells. Remarkably, these genes also showed higher expression in MCF7-TamR cells than in MCF7 cells. Our results not only validated the accuracy of HiSIF, but also demonstrated the gained looping events further enhance the gene expression in the resistant cells. We then performed the KEGG pathway analysis and found that the upregulated genes associated with gained loops in MCF7-TamR cells were significantly enriched with three signaling pathways: ECM receptor interaction, regulation of actin cytoskeleton, and focal adhesion (Additional file 1: Fig. S10) [45]. Furthermore, these genes were the core components of the signaling pathway networks (Fig. 5d) by using GeneMANIA, an online tool to perform functional network integration for gene prioritization [46]. We thus identified two major superfamily genes with gained loops, integrin superfamily (ITGA3, ITGA5, ITGA6, ITGB1, ITGB2, and ITGB8) and VEGF superfamily (VEGFA, VEGFC). Fibronectin 1 (FN1) is also a major component interacted with integrin and VEGF. We finally performed the K-M relapse-free survival analysis and found that higher expression of CD9 (Fig. 5e) and additional 20 genes, including ACSL3, BDKRB2, BIRC7, CEACAM5, DDX19B, DECR1, GDPD5, HEBP2, MGST3, MTHFD2L, NDUFV2, NR1D1, NUDT9, RRBP1, SLC12A8, SLC39A7, STYK1, TMEM184B, TRMT61B, and TTC13 (Additional file 1: Figs. S11–17-left column), which have gained loops in the resistant cells, were able to predict worse survival probabilities in endocrine-treated patients [47], but not in patients without endocrine therapy (Fig. 5f and Additional file 1: Figs. S11–17-right column), suggesting the genes with enhanced loops can be used for prognostic signatures for measuring the outcome of the endocrine treatment.

## Discussion

In this study, we have developed a novel computational method, HiSIF, to identify distinct classes of chromatin interactions from all-all interactions in Hi-C data. In addition, we demonstrated the performance and applicability of our HiSIF method using Hi-C and in situ Hi-C data publicly available [5, 35] as well as in situ Hi-C data generated by our own in breast cancer tamoxifen-sensitive and resistant cells (see the “Results” section).

Current computational efforts in analyzing Hi-C data are mainly focused on the identification of mega-base-size domains, i.e., TADs and compartments, and thus those large-scale structural methods are very limited when used to interpret the underlying functional and mechanistic relationship of 3D chromatin structure and individual gene regulation. Although HiCCUPS [5] and Fit-Hi-C [13] were designed for detecting chromatin interacting pairs, they have been reported to call only a small fraction of true-positive interactions [48]. Further, the accuracy of HICCUPS heavily relied on ultra-depth sequencing Hi-C data; the statistical confidence estimate assigned by Fit-Hi-C to intra-chromosomal contacts is in mid-range [5, 13]. In contrast, our HiSIF statistically models the distributions of the frequency and genomic distance of pairs of ligated DNA segments and defines two inter-dependent measures, FTR and FDR, used for identifying SIFs. By optimizing these two measures, HiSIF is able to identify chromatin interaction fragments at a relatively higher resolution with a relatively lower sequencing depth.

Remarkably, three enhanced looping genes in resistant breast cancer cells, ACP1, HECTD1, and MBIP, identified and validated by HiSIF and 3C/RT-qPCR (Fig. 4a–c), have been previously shown to be involved in breast cancer cell transformation and progression. For example, tyrosine-protein kinase receptor (EPHA2) is a prominent substrate of ACP1 and its kinase activity regulated by ACP1 can induce the cell transformation in breast cancer [49]. HECTD1 ubiquitinates phosphatidylinositol-4-phosphate 5-kinase type 1 gamma (PIP5K1C) at lysine 97 resulting in PIP5K1C degradation, consequently leading to focal adhesions dynamics and cell migration in breast cancer cells [50]. MBIP has also been found to contribute to the development of breast cancer in a genome-wide pathway analysis [51].

Intriguingly, two superfamilies, integrins and VEGFs, identified with gained loops in resistant breast cancer cells in this study have previously been shown to be functionally linked to breast cancers progression, metastasis, and treatment resistance. ITGB1, ITGB2, and ITGB8 belonging to integrin β subunits are the key components of the cell migration machinery and their major cellular receptors facilitate cell-extracellular matrix (ECM) adhesion. Indeed, ITGB1 is essential for cancer chemo-resistance and metastasis mediated by aberrant actin-bundling protein in breast cancer stem cells [52], and its signaling foster resistance to inhibitors of HER2 and PI3K in HER2+ breast cancer [53, 54]. Upregulation of ITGB2 promotes the migration and invasion in breast cancer [55].

VEGFs, the important signaling proteins for vasculogenesis and angiogenesis act as autocrine signaling molecules to stimulate the tumor growth and invasion [56–58] and induce epithelial-mesenchymal transition (EMT) to drive metastases of breast cancer [59]. VEGFs can also function like chemokine to recruit regulatory T cells resulting in the abasement of anti-tumor immune response and enhancement of tumor progression [60]. Bevacizumab and aflibercept have been approved by FDA for VEGF-targeted therapy for oncology [61]. Our findings suggest that oncogenic activities of both superfamilies may be regulated through promoter-distal looping mechanism.

Although FN1 and CD9 are not part of the above two superfamilies, notably, both binds or interacts with integrins and together have been demonstrated to functionally and mechanistically drive breast cancer progression and metastasis. For example, overexpression of FN1 is associated with tumor aggressiveness, metastasis, and poor prognosis of breast cancer [62, 63]. EMT transition can be induced by FN1 in human breast cancer MCF7 cells [64]. Overexpression of CD9 has been found to be related to invasiveness and metastases in breast cancer cells [65, 66]. Our study further identified a higher interaction frequency of CD9 promoter-distal looping in resistant breast cancer cells and illustrated that such higher expression is evidently associated with lower survival probability in endocrine-resistant breast cancer patients (Fig. 5e, f). Future work may focus on characterizing how chromatin looping of FN1 and CD9 functionally and mechanistically contributes to ERα + breast cancer resistant to the endocrine therapy.

## Conclusions

In summary, we developed a statistically modeled and rigorously tested method, HiSIF, for the functional analysis of 3D chromatin structure and specific gene regulation. With HiSIF, we identified two enriched signaling pathways, integrins and VEGFs, showing enhanced promoter-distal loops in endocrine-resistant breast cancer cells. Higher expression of 21 genes is associated with worse relapse-free survival in endocrine-treated patients, suggesting they might be used for prognostic signatures for measuring the outcome of the endocrine treatment and developing therapeutic targets. HiSIF is applicable for any Hi-C data in any normal and diseased cells or tissues.

## Supplementary information

**Additional file 1.** Supplementary methods, figures and tables.

**Additional file 2.** Significant interacting fragments called by HiSIF in GM12878 cells Replicate 1.

**Additional file 3.** Significant interacting fragments called by HiSIF in GM12878 cells Replicate 3–9.

**Additional file 4.** Significant interacting fragments called by HiSIF in K562 cells.

**Additional file 5.** Significant interacting fragments called by HICCUPS in K562 cells.

**Additional file 6.** Significant interacting fragments called by Fit-Hi-C in K562 cells.

**Additional file 7.** Gained promoter-distal loops in MCF7-TamR cells.

**Additional file 8.** Up-regulated DEGs with Gained promoter-distal loops in MCF7-TamR cells.

## Data Availability

Publicly available human Hi-C datasets representing different experimental protocols and sequencing depths were used for training and testing HiSIF including Hi-C data in MCF10A and MCF7 cells [36], hESC cells [35], in situ Hi-C data in GM12878 cells and K562 cells [5]. Application of HiSIF in Hi-C data in breast cancer sensitive and resistant MCF7 and MCF7-TamR cells were from our previous study [37]. The source codes and compiled tool for HiSIF can be accessed from https://github.com/yufanzhouonline/HiSIF [40].
