# Supplementary material for: Modeling and analysis of Hi-C data by HiSIF identifies characteristic promoter-distal loops
Source: Genome Med. 2020 Aug 12;12:69. doi: 10.1186/s13073-020-00769-8 (PMC7425017; doi:10.1186/s13073-020-00769-8)
Supplement: Supplementary file 1 — Additional file 1. Supplementary methods, figures and tables. [file 13073_2020_769_MOESM1_ESM.docx]

**Additional file 1: Supplementary Methods, Figures and Tables**

**Supplementary Methods**

**Quality Control of Hi-C, TCC and in situ Hi-C data**

We used ten fragments numerated as F1-F10 to demonstrate the Quality Control module. In a Hi-C, TCC or in situ Hi-C protocol, the genome is cut into HindIII or MboI enzyme-digested fragments (**Supplementary Figure S1 – left**). Any paired-end (PE) reads uniquely mapped to two particular restriction fragments were termed as Ultrasonic Fragments (USFs), as shown in Green, Light Blue and Orange. Three USFs were mapped to F2 and F9, one USF to F3 and F7 and another USF to F5 and F6. A view of Hi-C interactions with four restriction fragments was shown in Figure 1A and with eight restriction fragments in **Supplementary Figure S1 -- right**. In the first step, we removed any USFs mapped to the same restriction fragments. These fragments are marked in Red and further categorized into self-loops and dangling ends depending on the direction of mapping. Next, we removed re-ligation events marked in Yellow which were mapped to the nearest restriction fragment. To remove re-ligation USFs, we applied a 20Kb and 3Kb distance cut-off threshold between any two PE reads for HindIII and for MboI restriction enzymes respectively. The chosen thresholds are based on the distribution of the length of the enzyme digested restriction fragments (**Supplementary Figure S2**), where most of the restriction fragment sizes for HindIII are less than 20Kb and for MboI less than 3Kb. Therefore, it was reasonable to assume there would be at least one restriction fragment occurring for any two PE reads less than these two limits between them. Theoretically, any single read mapped in a Hi-C library should be in the vicinity of a particular restriction fragment, thus we removed any reads beyond the 500 bp distance from the restriction enzyme cutting sites as previously explained. The remaining USFs were subject to the Classification module to separate random and proximate ligation events.

**Mathematical derivations of recursive relation for the Poisson mixture model**

In the following section, we show the derivation of the likelihood function and the Expectation Maximization (EM) recursive relations for the Poisson mixture model. The problem of separating the random-ligation events from the proximate-ligation events can be specified as following: what are the two Poisson components (one for the random and one for the proximate) that represent the total paired end fragment distribution in the vicinity of enzyme digested fragments? If this double Poisson is an adequate model for describing this USF distribution, the one-dimensional Poisson Mixture Model (PMM) is well suited to the problem. It is evident that, by adding more Poisson components to the mixture model users can improve the quality of fit, but defining a physical meaning to each component is clearly impossible. A decision need to be taken to derive the number of Poisson components in the mixture by trading off quality of fit against the number of introduced free parameters. We use the Bayesian Information Criterion (BIC) to determine how many mixture components we should use in the original data. The BIC is defined as

$BIC=-2logL_{max}+klogM$ S1

here $k$ represents the number of free parameters. Bellow we describe how to fit a multi-component Poisson mixture model to a one-dimensional distribution of data. The notation follows that the data are to be modeled by a mixture of $K$ Poisson fit to the distribution of $N$ data points. The subscript $l$ cycles through $N$ and $k$ cycles through $K$, and we use $\lambda_{k}$ and $\omega_{k}$ to denote the location (Poisson mean) and weight of each Poisson component. Data points are represented by $d_{l}$. For derivation, we denote the parameters ($\lambda_{k}$ and $\omega_{k}$) collectively by $\theta$. Then the log likelihood of the parameters of equation S1 given the data is

$l\left( D:\theta\right)=\sum_{l=1}^{N} log\sum_{k=1}^{K} \omega_{k}g\left( D_{l}:\lambda_{k} \right)$ S2

The EM algorithm provides an efficient way to get the maximum-likelihood estimators. After some algebra, we can derive a set of relations that lead to the maximum likelihood. We can write the corresponding Poisson probability density function as

$g\left( x:\lambda\right)=\frac{\lambda^{x}}{x!}exp\left( -\lambda\right)$ S3

The joint probability of selecting a component ($\omega_{k}$) and selecting an observation from that component ($g\left( D_{l}:\lambda_{k} \right)$) is given by:

$q\left( k,l \right)=\omega_{k}g\left( D_{l}:\lambda_{k} \right)$ S4

By using Bayes' theorem, we can write the conditional probability of selecting component $k$ given the observation $D_{l}$ as:

$p\left( k\vee l \right)=\frac{q\left( k,l \right)}{\sum_{k=1}^{K} q\left( k,l \right)}$ S5

To start the EM algorithm users has to assume the initial mixture parameters and Poisson means for each mixture. The first step is called the “E” step where we try to find an upper bound $b$ on $l$, in this derivation we use Jensen’s inequality to derive the upper bound. The second step is the “M” step where, we find parameter estimates which maximize the bound. Using the fact that $p(k|l)$ in S6 sum up to 1 and Jensen's inequality we can bound the liklihood as:

$l\left( D:\theta\right)=\sum_{l=1}^{N} log\sum_{k=1}^{K} q\left( k,l \right)\geq\sum_{l=1}^{N} \sum_{k=1}^{K} p\left( k,l \right)log\frac{q\left( k,l \right)}{p\left( k\vee l \right)}=b\left( \theta\right)$ S6

By expanding the log and distribute $q(k,l)$ we can get the first term of the above S7 as:

$Q\left( \theta\right)=\sum_{l=1}^{N} \sum_{k=1}^{K} p\left( k\vee l \right)logq\left( k,l \right)$ S7

Using above S8 we can derive the updated mixture parameters for the maximum log likelihood as

$\frac{\partial Q\left( \theta\right)}{\partial\lambda_{k}}=\frac{\partial Q\left( \theta\right)}{\partial\omega_{k}}=0$ S8

For the Poisson mean we can derive:

$\lambda_{k}=\frac{\sum_{\text{l=}1}^{N} p\left( k\vee l \right)D_{l}}{\sum_{\text{l=}1}^{N} p\left( k\vee l \right)}$ S9

and for mixing probabilities we can derive

$\omega_{k}=\frac{1}{N}\sum_{\text{l=}1}^{N} p\left( k\vee l \right)$ S10

**Instruction on how to run HiSIF**

HiSIF is written in C and C++, and the source codes can be accessed from https://github.com/yufanzhouonline/HiSIF. To run HiSIF, we need two input files: the filtered PE read file in text format and the enzyme digested cutting site file in bed format. For restriction enzyme HindIII and MboI, we provide appropriate bed files in the repository. The filtered PE reads file is a six-column text file, where first to last columns stand for chromosome one, mapping position one, strand one, chromosome two, mapping position two, strand two respectively. Chromosomes are labeled from 1 to 24, where 23 and 24 are short for Chromosome X and Y respectively. The label of strand is 1 for positive strand and 0 for the negative strand. All the preprocessed data need to be in the above order and there is a Perl script available for the preprocessing in the repository. If the user needs to map and filter their own data, we recommend using hiclib up to the fragment level filtering. To convert filtered HDF5 file into HiSIF text file, a simple python script is also available in the repository. Users need hg19 or hg18 genome directories to find the cutting site lengths and full path need to be passed as a command line argument for HiSIF executable. Only other parameters need to pass are Poison means for random, proximate ligations average read length of the original FASTQ file and the maximum length of the enzyme digested restriction fragment, which can be found in **Supplementary** **Figure S2**.

The output of the HiSIF is an eight-column text file, where:

- 1^st^ column: fragment 1 chromosome number
- 2^nd^ column: fragment 1 start position
- 3^rd^ column: fragment 1 end position
- 4^th^ column: fragment 2 chromosome number
- 5^th^ column: fragment 2 start position
- 6^th^ column: fragment 3 end position
- 7^th^ column: FTR value
- 8^th^ column: FDR value

**Supplementary Figures**


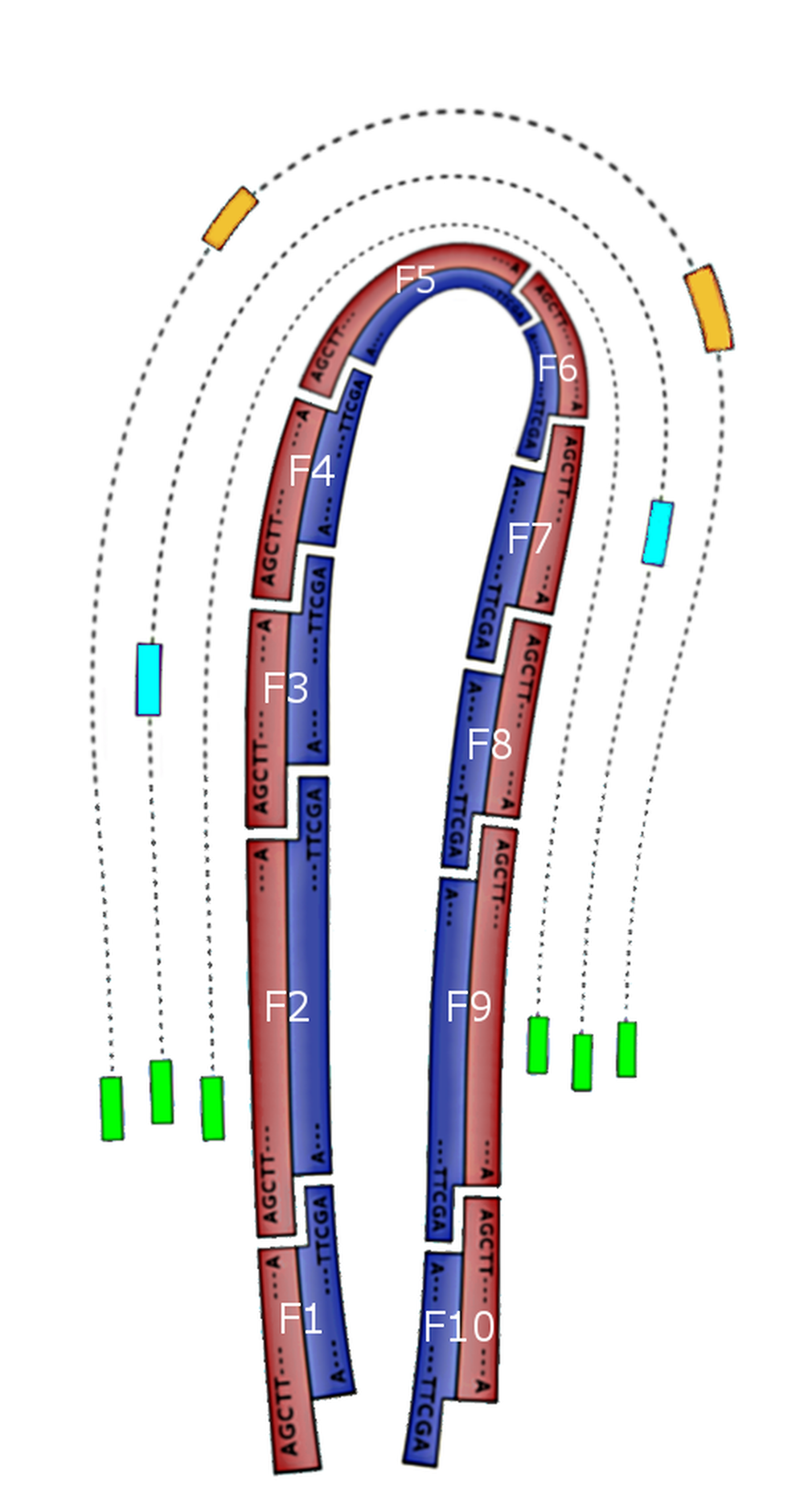


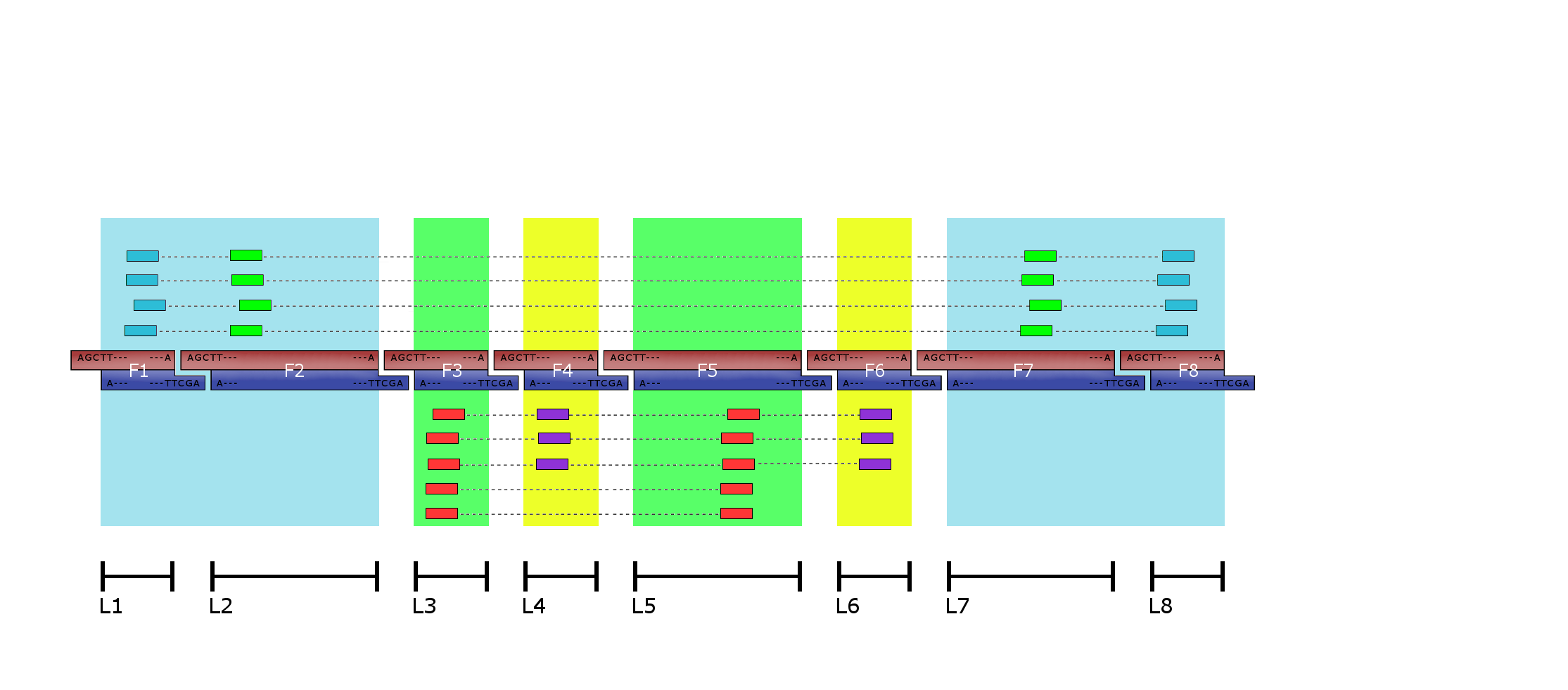


|  |  |  |
| --- | --- | --- |
| **Figure S1**. Illustration of chromosomal interactions with the USF distribution in the vicinity of HindIII digested restriction fragment cut sites. An example Hi-C interaction with USF and digested restriction sites (left). The green color fragments show a proximate-ligation interaction, blue color shows a random ligation, and the red shows a self-ligation. One dimensional view of Hi-C contacts and merging of interaction pairs (right). | | |

| 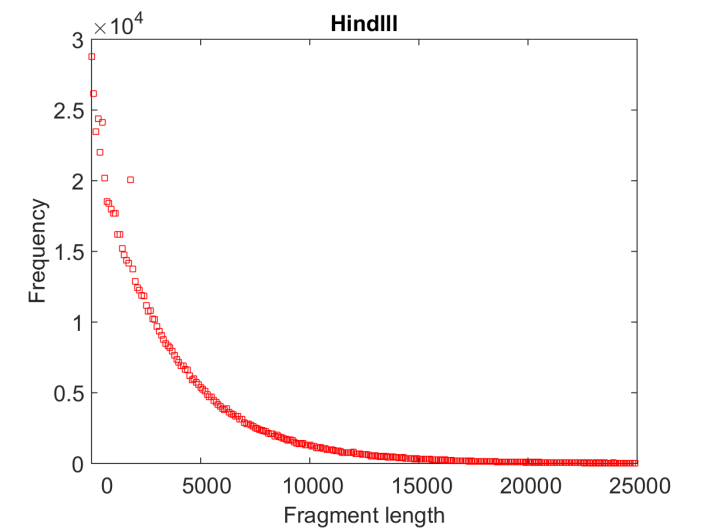 | 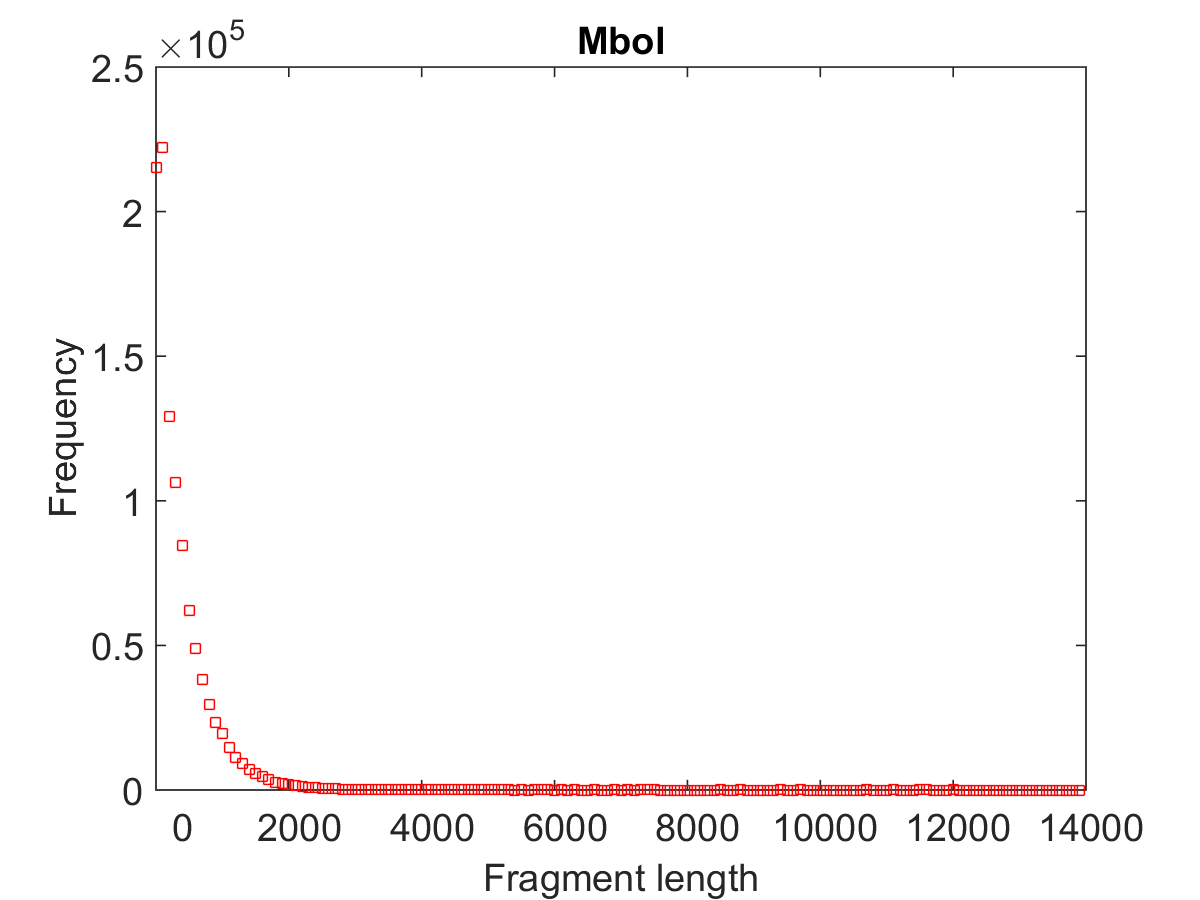 |
| --- | --- |

**Figure S2**. Enzyme digested DNA fragment lengths for HindIII (left) and MboI (right) to determine the re-ligation threshold.


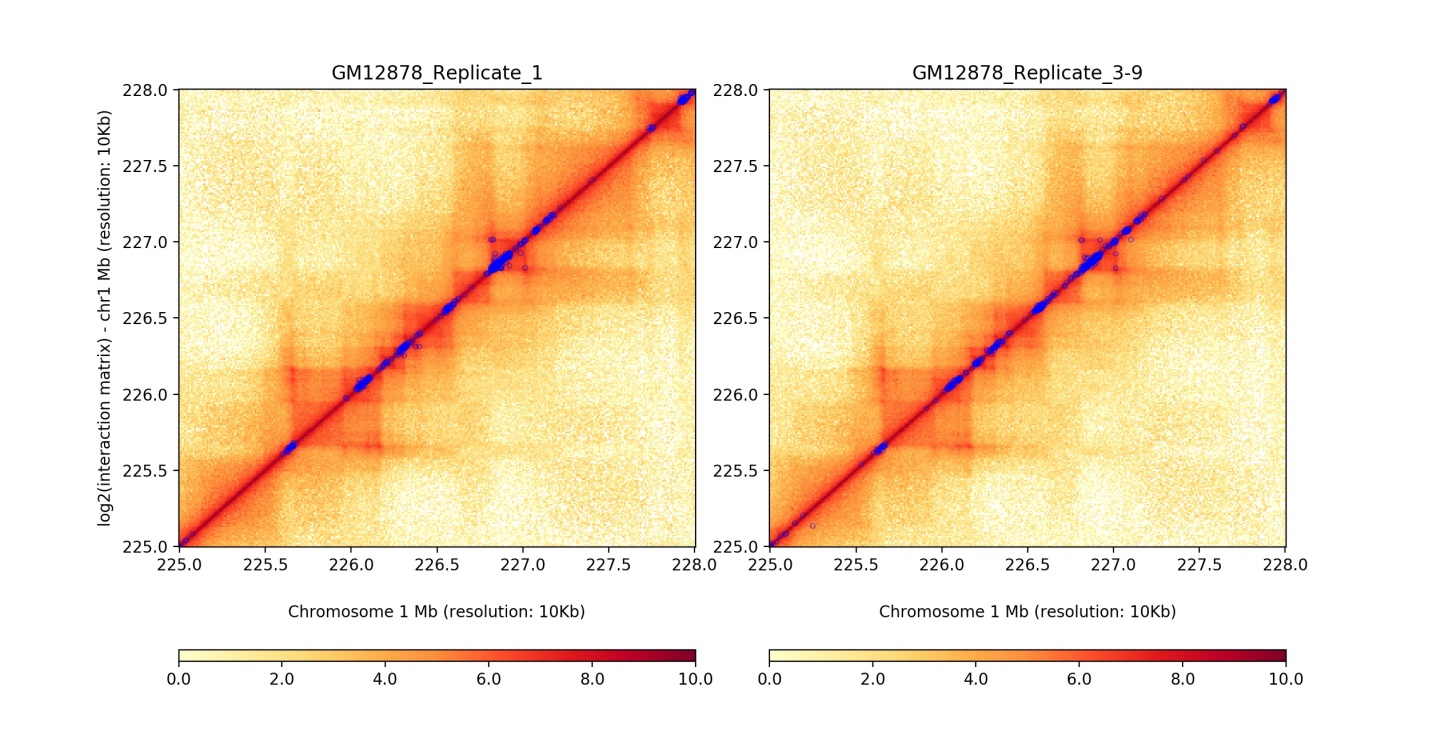


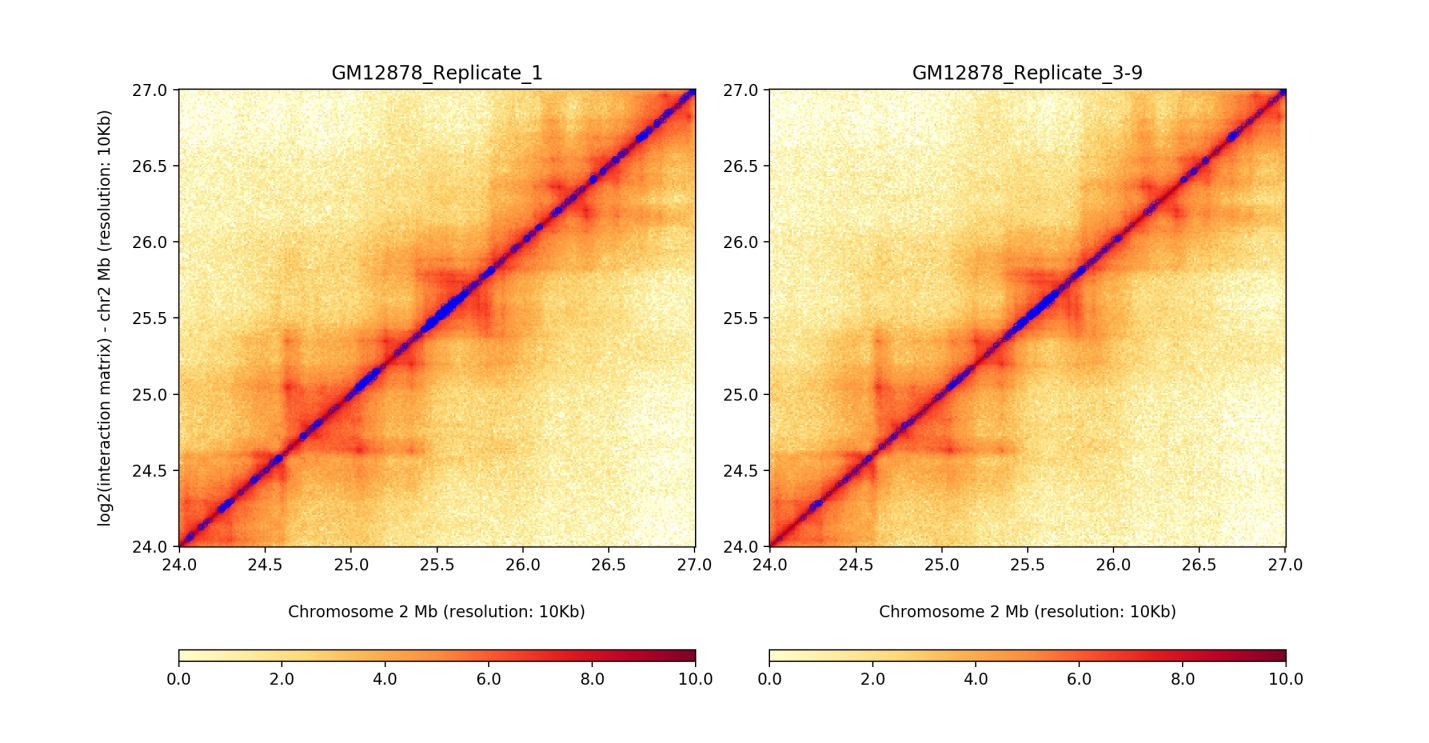


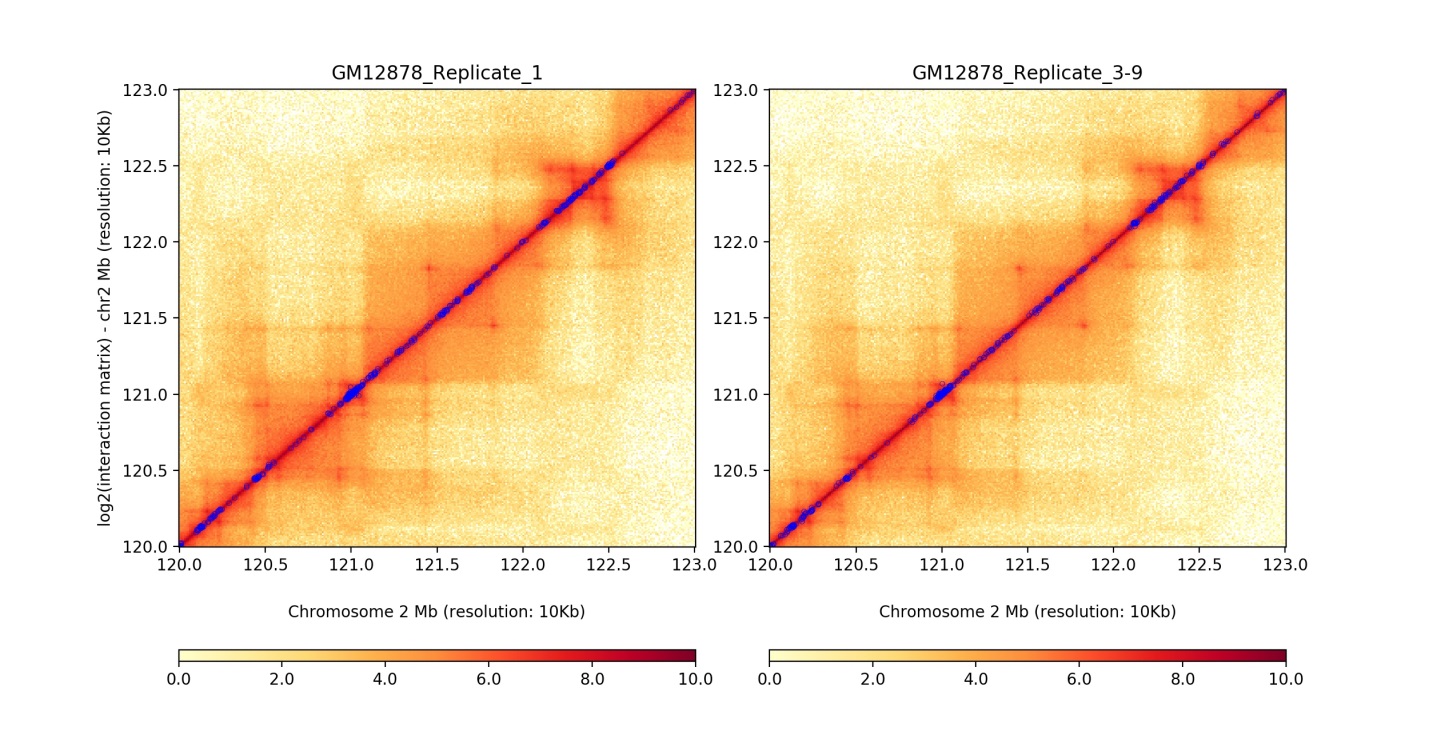


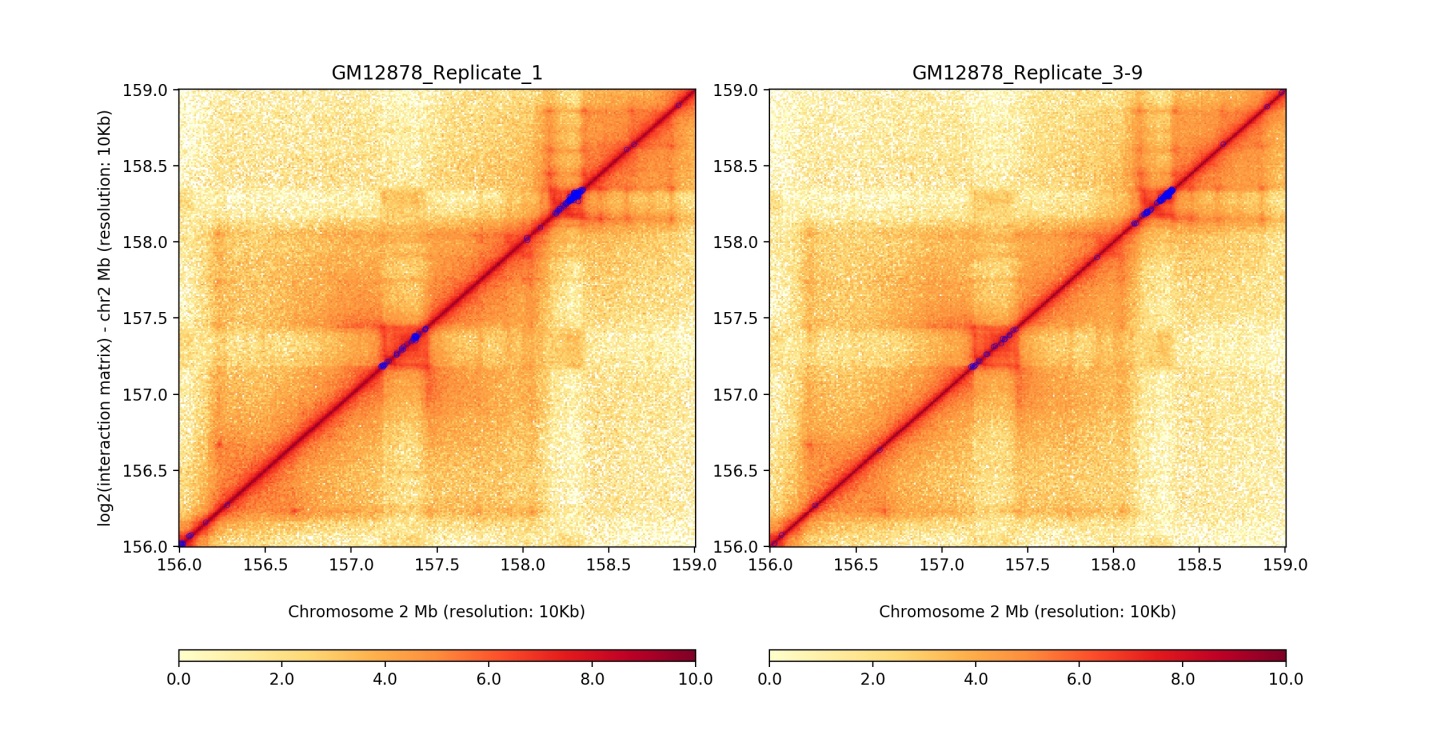


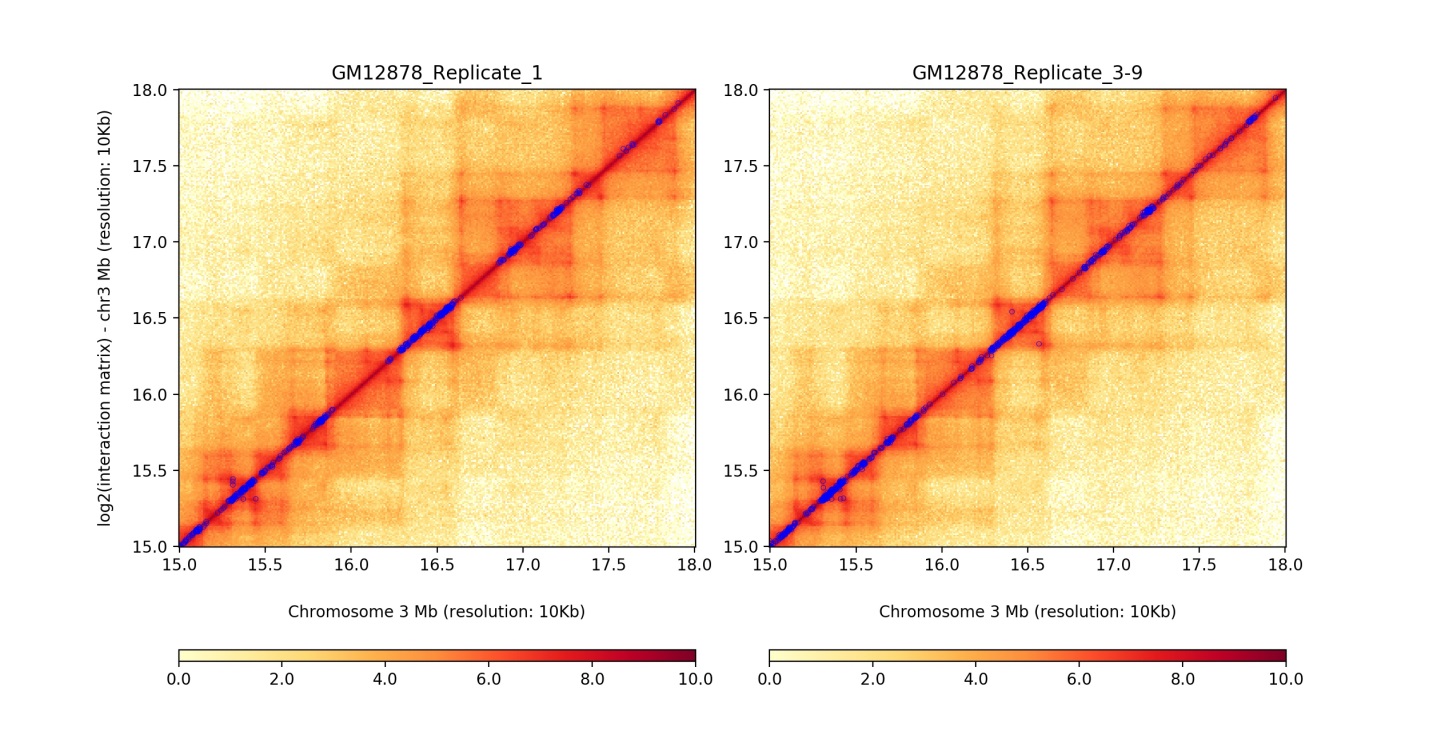


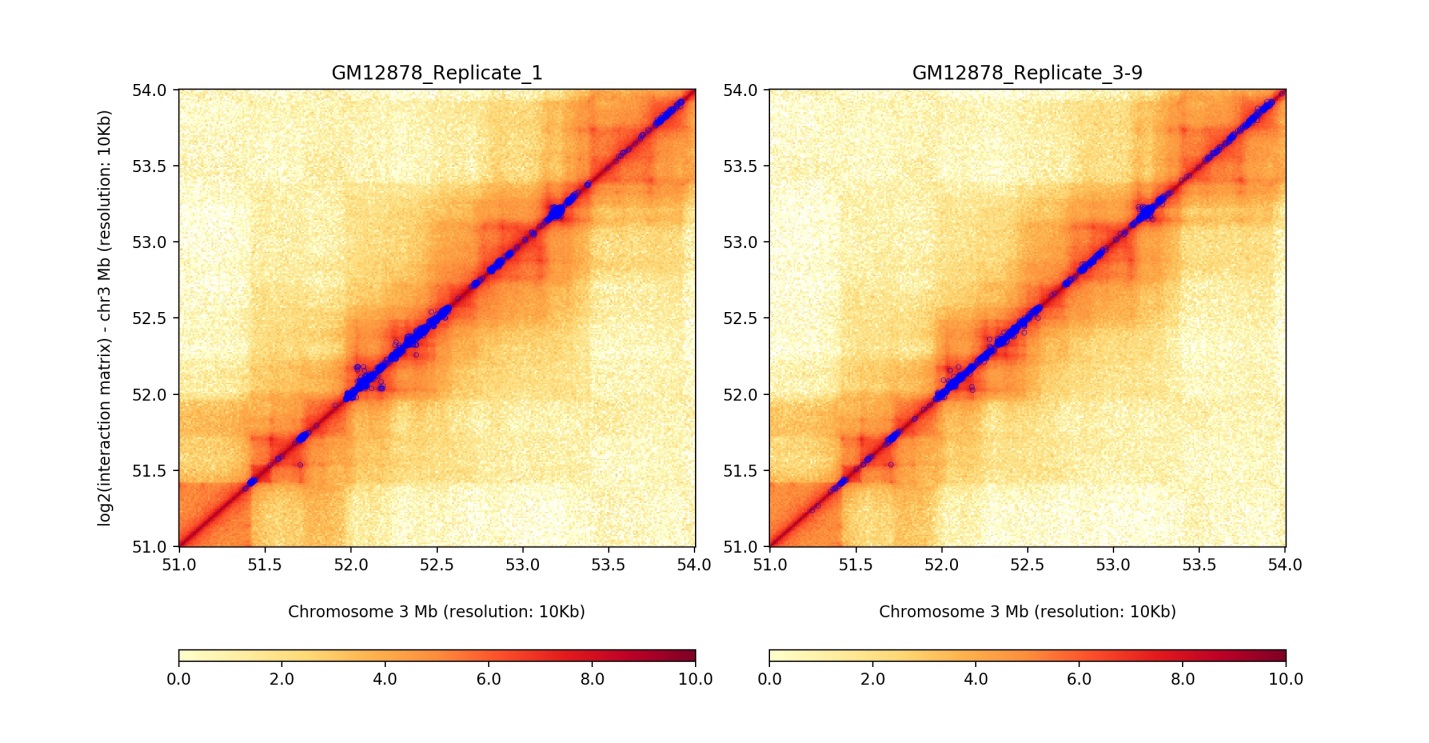


**Figure S3**. Screenshots of HiSIF loops on heatmap of GM12878 cells. The GM12878 HiSIF loops were produced from GM12878 Hi-C data downloaded from GEO GSE63525 (Rao et al. 2014) with the threshold of 2 and FDR<0.1. The central points of HiSIF loop foci are labelled with blue circles. The left columns are GM12878 replicate 1, the right columns are combination of replicate 3-9. The figures were generated with 10K resolution for the loci as indicated using open-source visualization tool HiCPlotter (Akdemir et al. 2015).


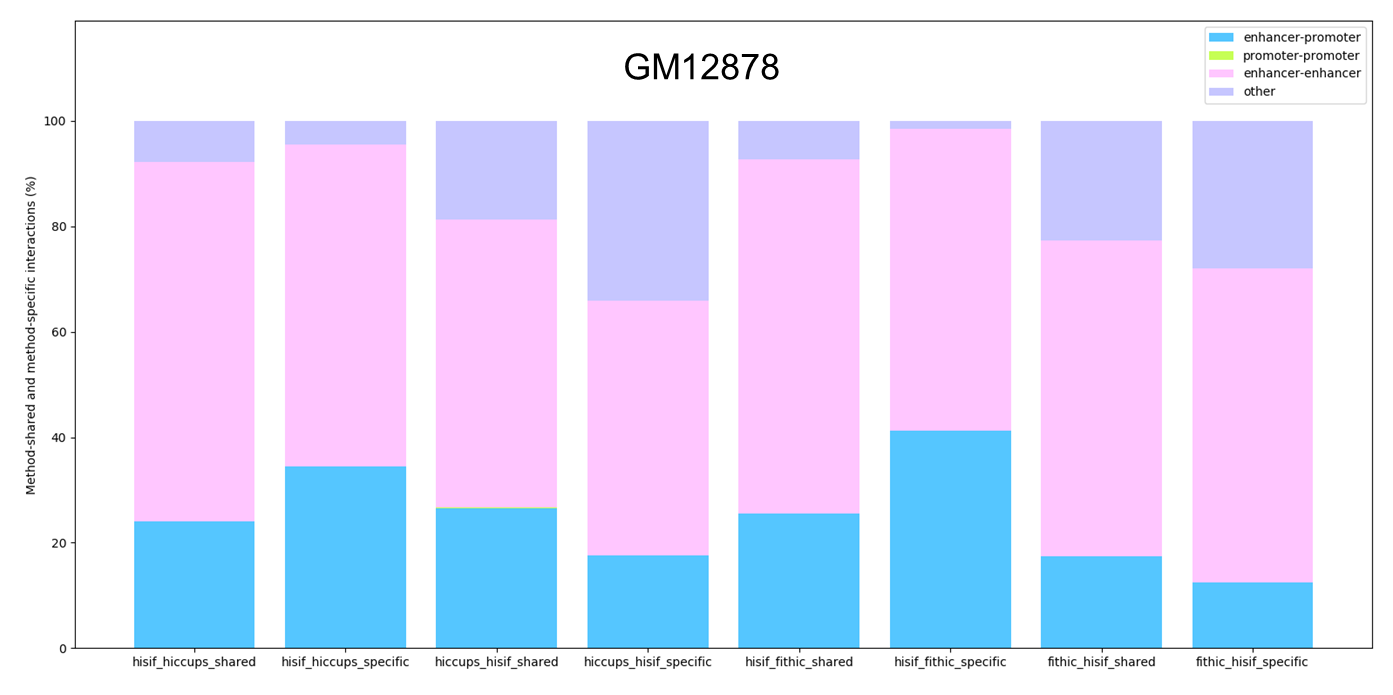


**Figure S4**. Method-shared and method-specific interactions for GM12878 cells. GM12878 Hi-C data were downloaded from GEO GSE63525 (Rao et al. 2014).

hisif_hiccups_shared: HiSIF shared loops comparing with HICCUPS.

hisif_hiccups_specific: HiSIF specific loops comparing with HICCUPS.

hiccups_hisif_shared: HICCUPS shared loops comparing with HiSIF.

hiccups_hisif_specific: HICCUPS specific loops comparing with HiSIF.

hisif_fithic_shared: HiSIF shared loops comparing with Fit-Hi-C.

hisif_fithic_specific: HiSIF specific loops comparing with Fit-Hi-C.

fithic_hisif_shared: Fit-Hi-C shared loops comparing with HiSIF.

fithic_hisif_specific: Fit-Hi-C specific loops comparing with HiSIF.


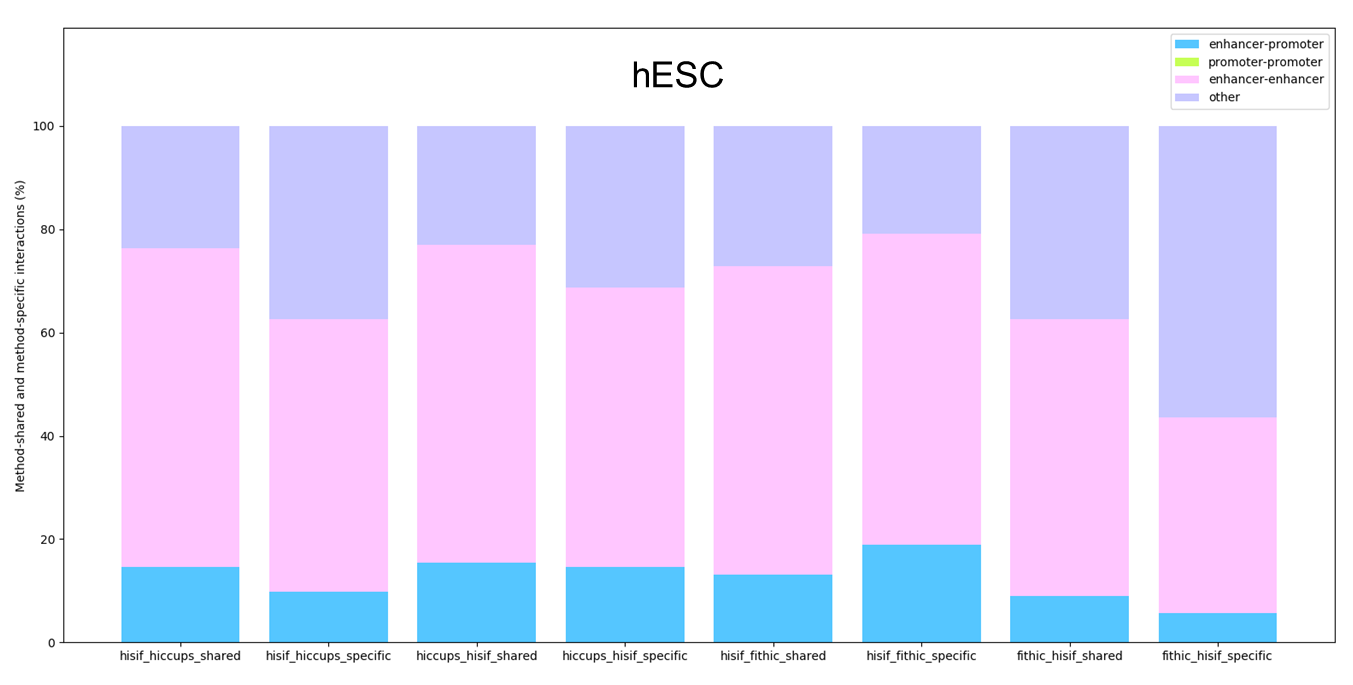


**Figure S5**. Method-shared and method-specific interactions for hESC cells. hESC Hi-C data were downloaded from GEO GSE52457 (Dixon et al. 2015).

hisif_hiccups_shared: HiSIF shared loops comparing with HICCUPS.

hisif_hiccups_specific: HiSIF specific loops comparing with HICCUPS.

hiccups_hisif_shared: HICCUPS shared loops comparing with HiSIF.

hiccups_hisif_specific: HICCUPS specific loops comparing with HiSIF.

hisif_fithic_shared: HiSIF shared loops comparing with Fit-Hi-C.

hisif_fithic_specific: HiSIF specific loops comparing with Fit-Hi-C.

fithic_hisif_shared: Fit-Hi-C shared loops comparing with HiSIF.

fithic_hisif_specific: Fit-Hi-C specific loops comparing with HiSIF.


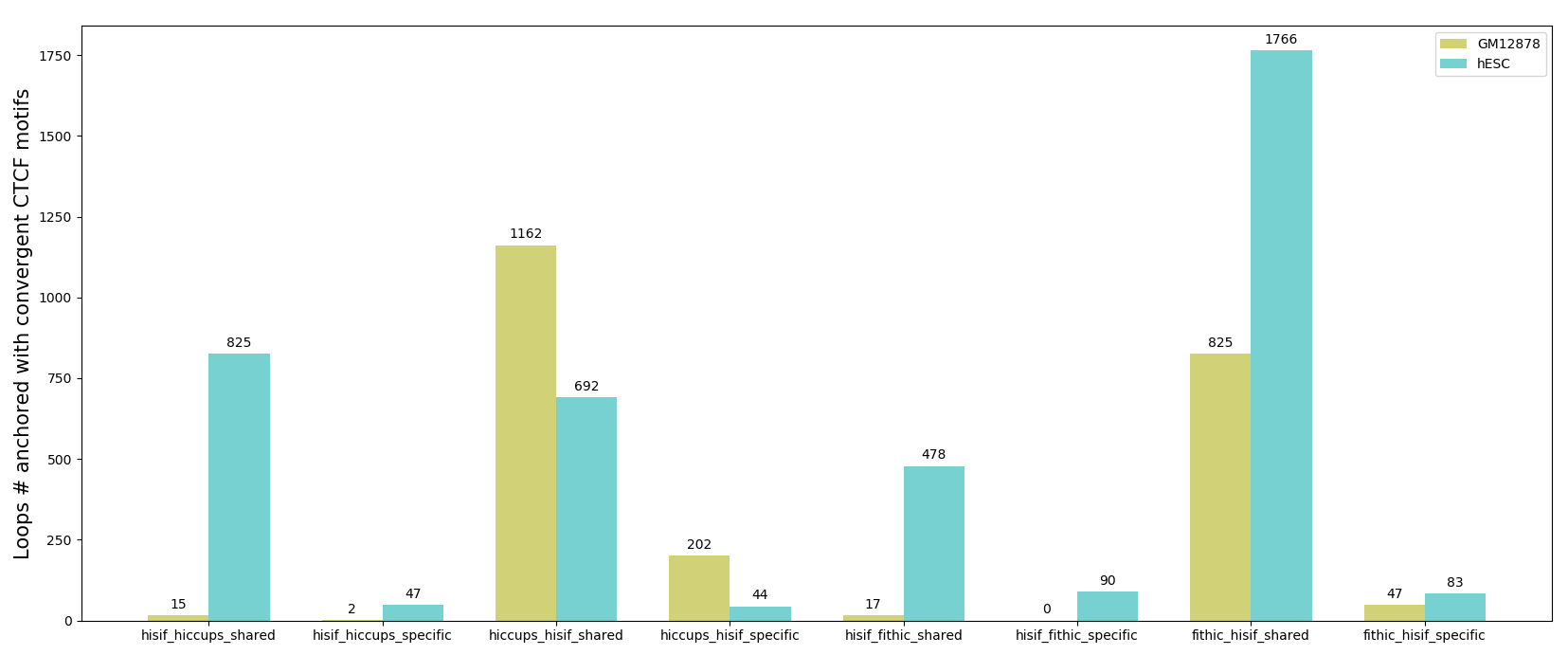


**Figure S6**. The numbers of loops anchored with convergent CTCF motifs. GM12878 Hi-C data were downloaded from GEO GSE63525 (Rao et al. 2014). hESC Hi-C data were downloaded from GEO GSE52457 (Dixon et al. 2015).

hisif_hiccups_shared: HiSIF shared loops comparing with HICCUPS.

hisif_hiccups_specific: HiSIF specific loops comparing with HICCUPS.

hiccups_hisif_shared: HICCUPS shared loops comparing with HiSIF.

hiccups_hisif_specific: HICCUPS specific loops comparing with HiSIF.

hisif_fithic_shared: HiSIF shared loops comparing with Fit-Hi-C.

hisif_fithic_specific: HiSIF specific loops comparing with Fit-Hi-C.

fithic_hisif_shared: Fit-Hi-C shared loops comparing with HiSIF.

fithic_hisif_specific: Fit-Hi-C specific loops comparing with HiSIF.


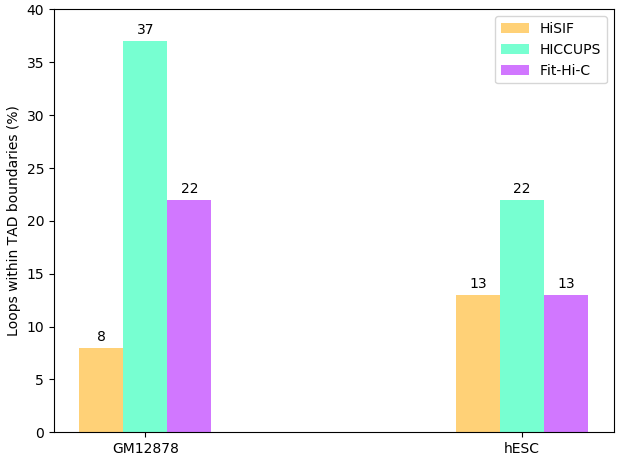


**Figure S7**. Loops within TAD boundaries in GM12878 and hESC cells. TADs (topologically associating domains) were called with insulation profiles with the parameters “-is 500000 -ids 200000 -im mean -bmoe 0 -nt 0.1 -v" (Crane et al. 2015) and with the bin size of 40K. GM12878 Hi-C data were downloaded from GEO GSE63525 (Rao et al. 2014). hESC Hi-C data were downloaded from GEO GSE52457 (Dixon et al. 2015).


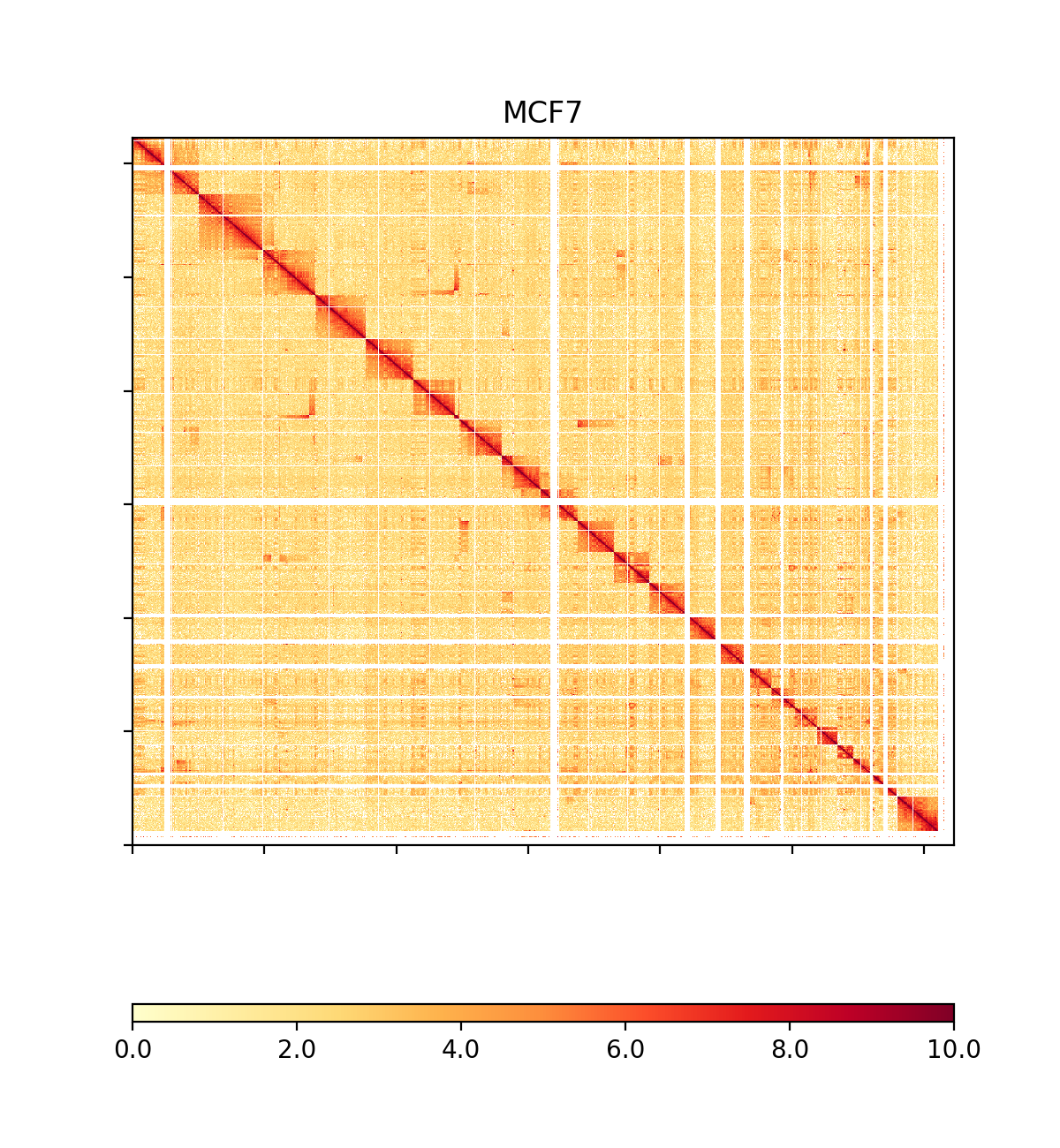


**Figure S8**. Heatmap of whole genome interactions of MCF7 cells. Whole genome interaction heatmap was generated with 1000K resolution using open-source visualization tool HiCPlotter (Akdemir et al. 2015).


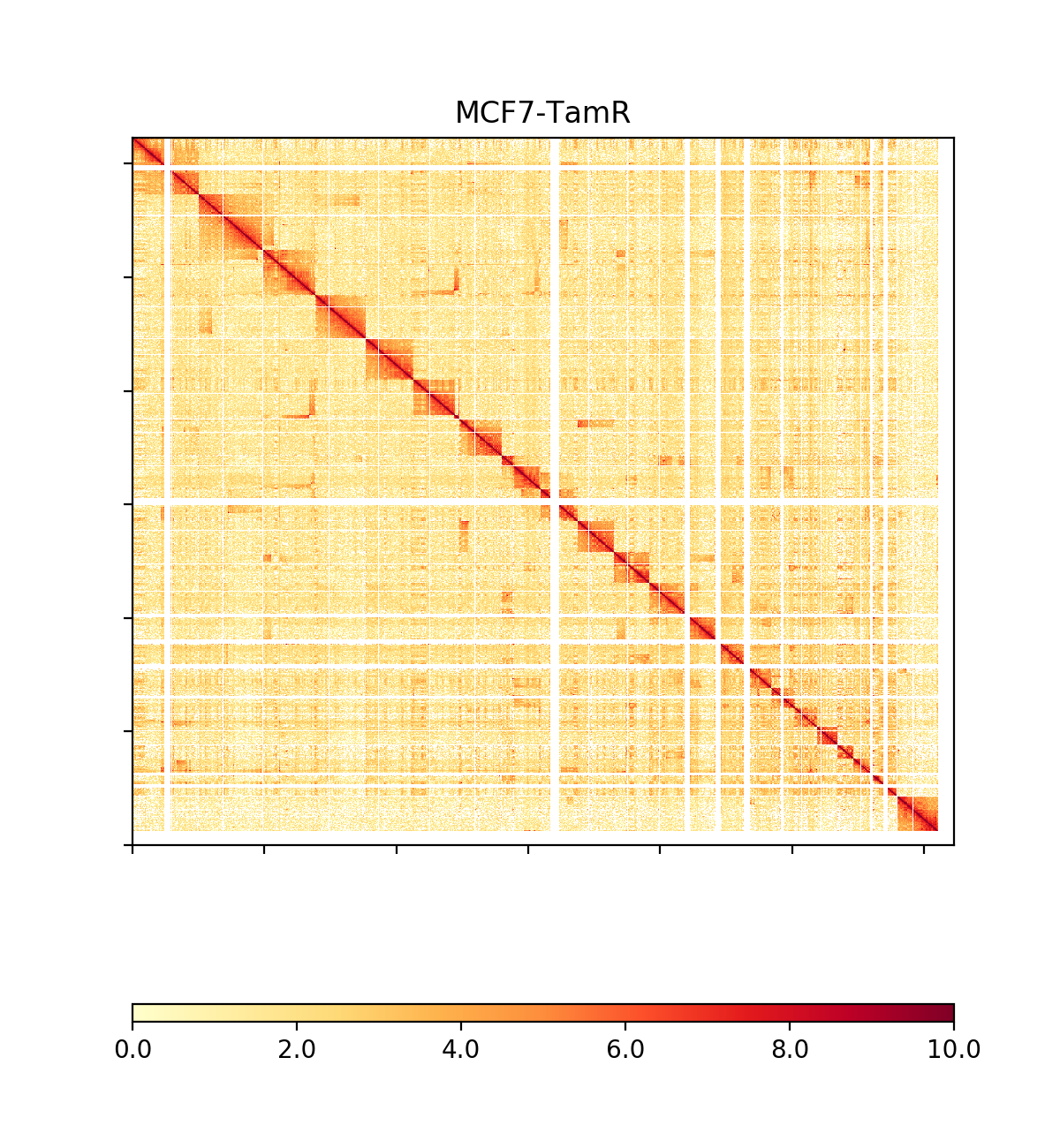


**Figure S9**. Heatmap of whole genome interactions of MCF7-TamR cells. Whole genome interaction heatmap was generated with 1000K resolution using open-source visualization tool HiCPlotter (Akdemir et al. 2015).

**
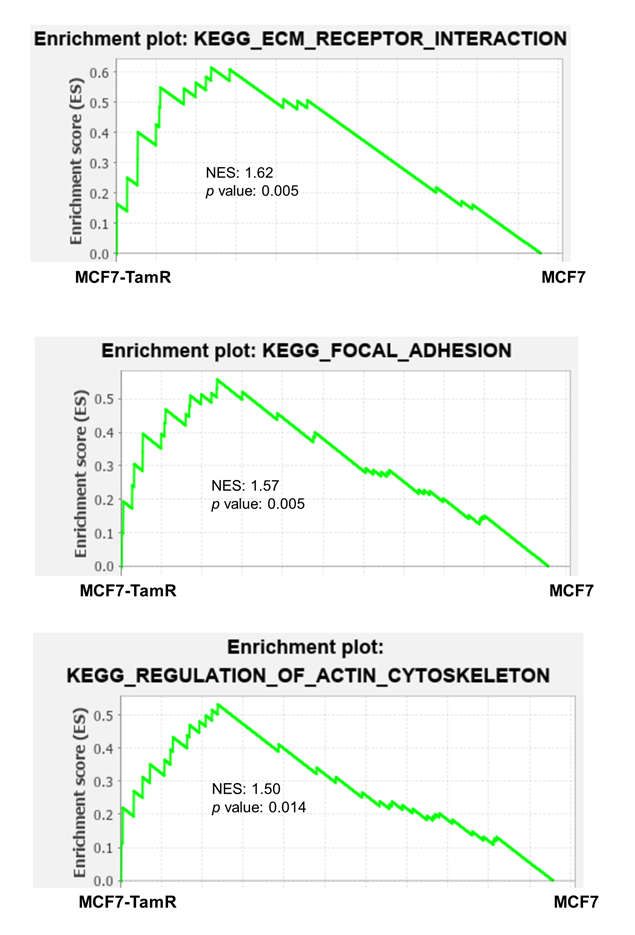
**

**Figure S10.** KEGG pathway analyses on the enrichment of the genes with gained PDLs in MCF7-TamR cells. NES: Normalized Enrichment Score.


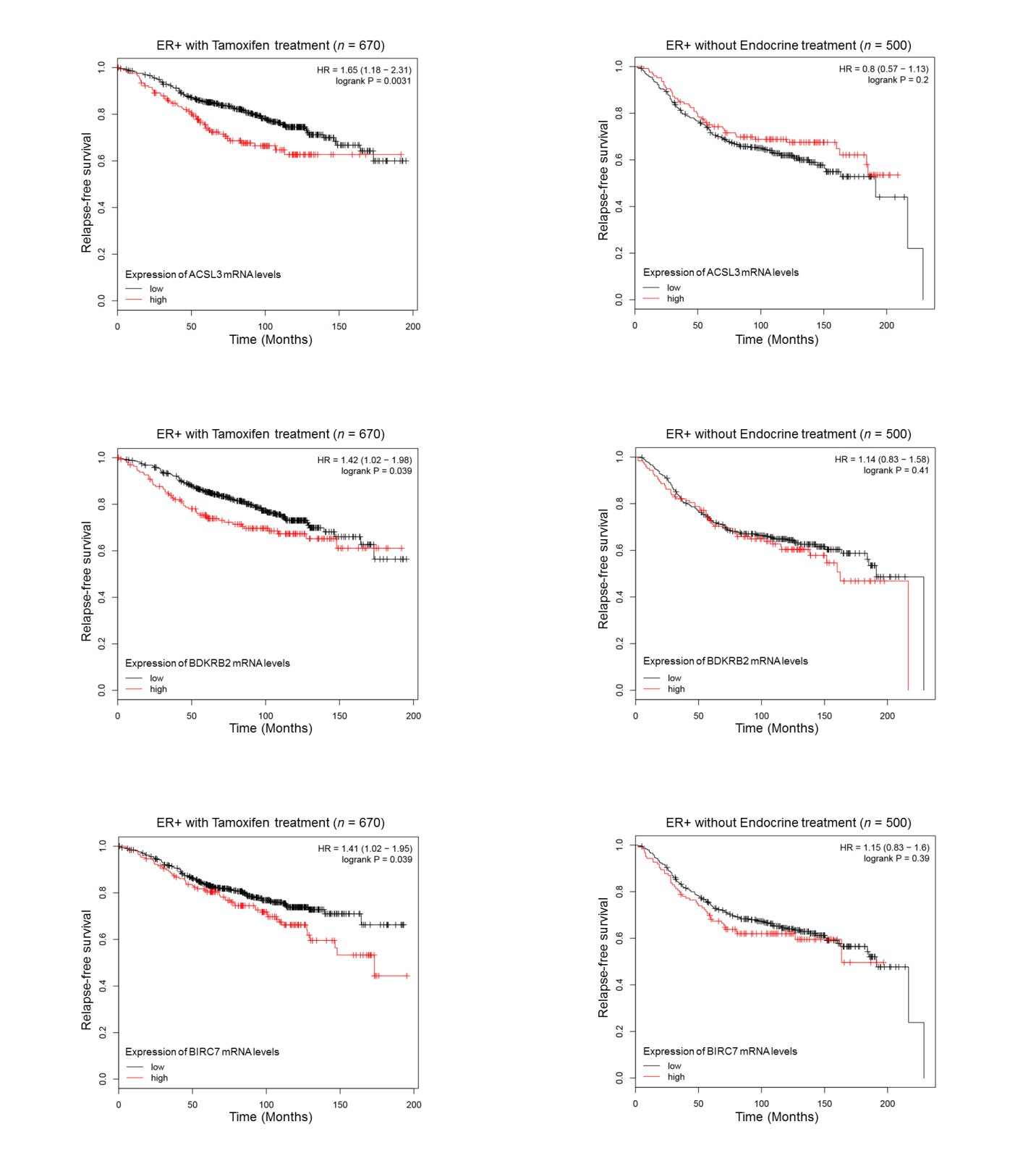


**Figure S11.** Relapse-free survival analysis of ACSL3, BDKRB2, BIRC7 in ERα+ patients. The *p* value was computed with the log-rank test. Analysis was referred to the published paper (Lanczky et al. 2016). HR: Hazard Ratio.


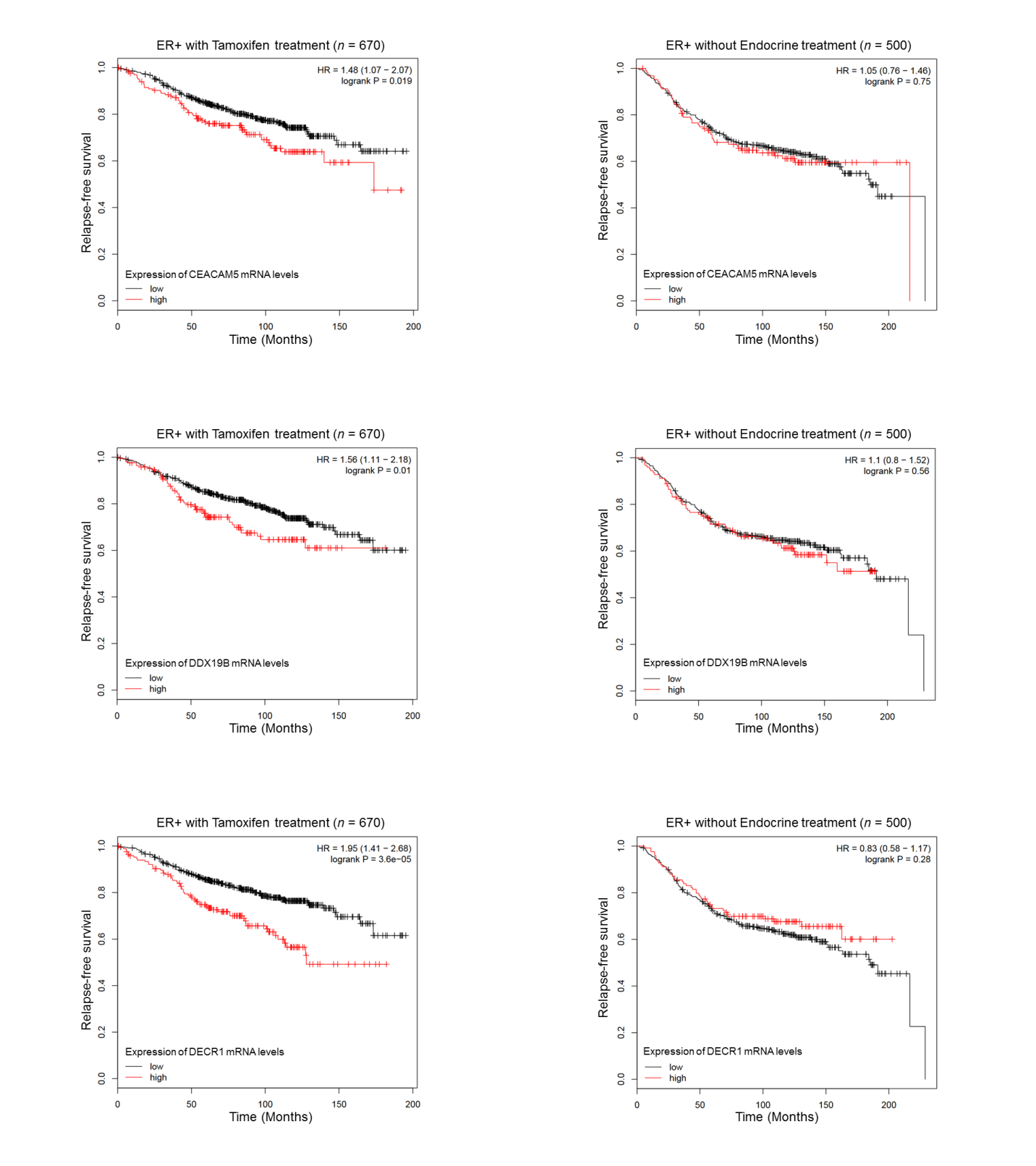


**Figure S12.** Relapse-free survival analysis of CEACAM5, DDX19B, DECR1 in ERα+ patients. The *p* value was computed with the log-rank test. Analysis was referred to the published paper (Lanczky et al. 2016). HR: Hazard Ratio.


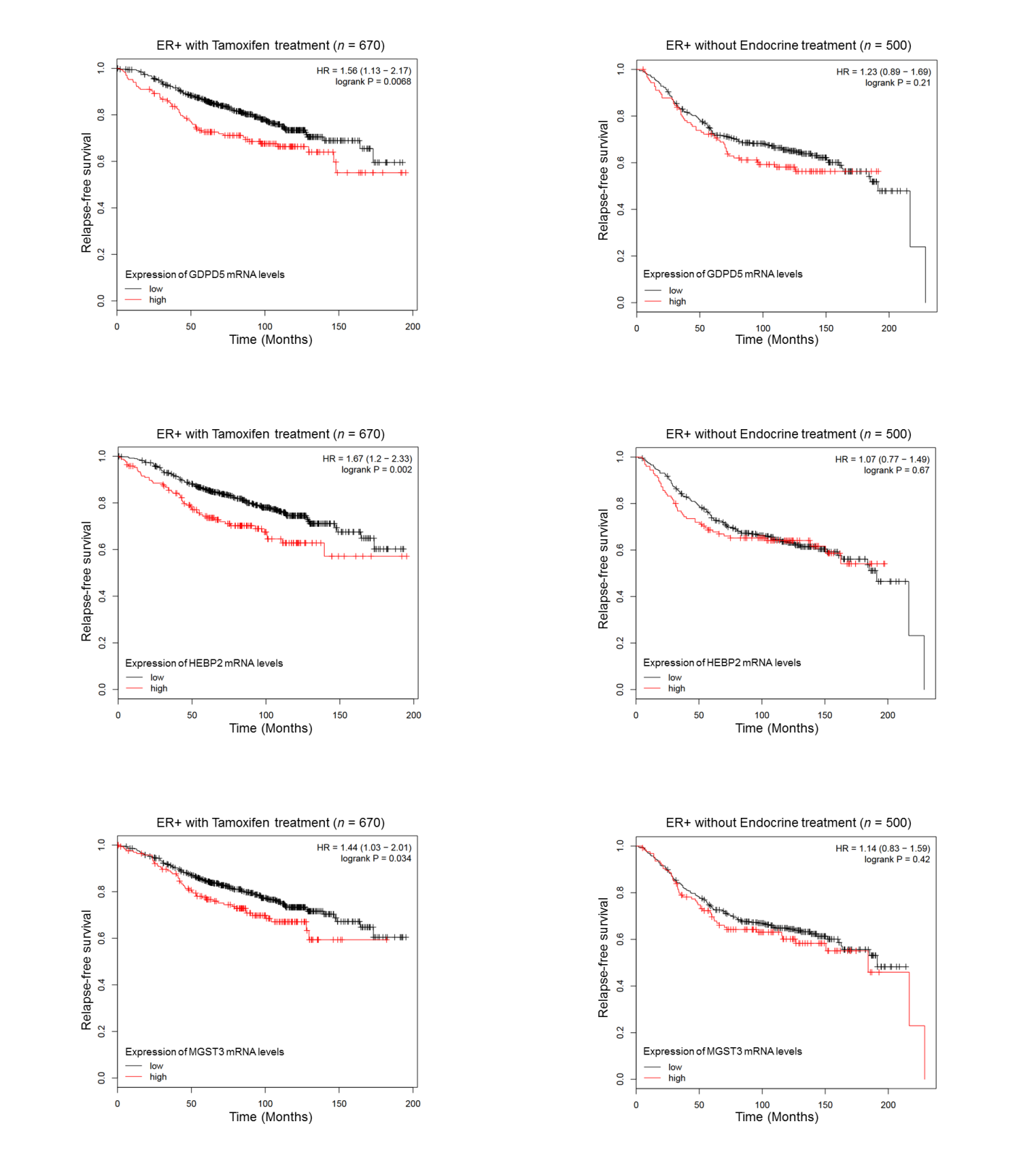


**Figure S13.** Relapse-free survival analysis of GDPD5, HEBP2, MGST3 in ER+ patients. The *p* value was computed with the log-rank test. Analysis was referred to the published paper (Lanczky et al. 2016). HR: Hazard Ratio.


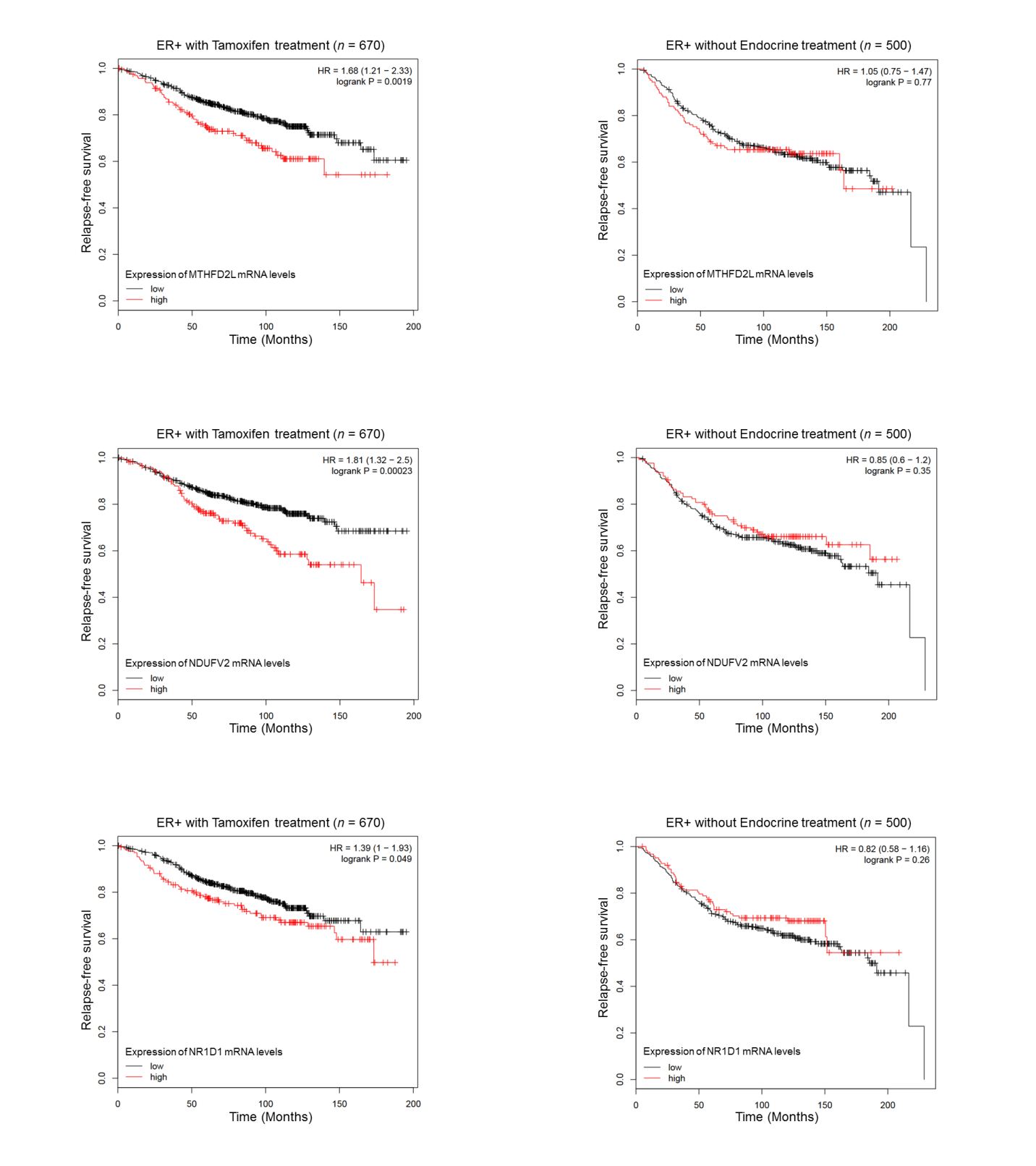


**Figure S14.** Relapse-free survival analysis of MTHFD2L, NDUFV2, NR1D1 in ER+ patients. The *p* value was computed with the log-rank test. Analysis was referred to the published paper (Lanczky et al. 2016). HR: Hazard Ratio.


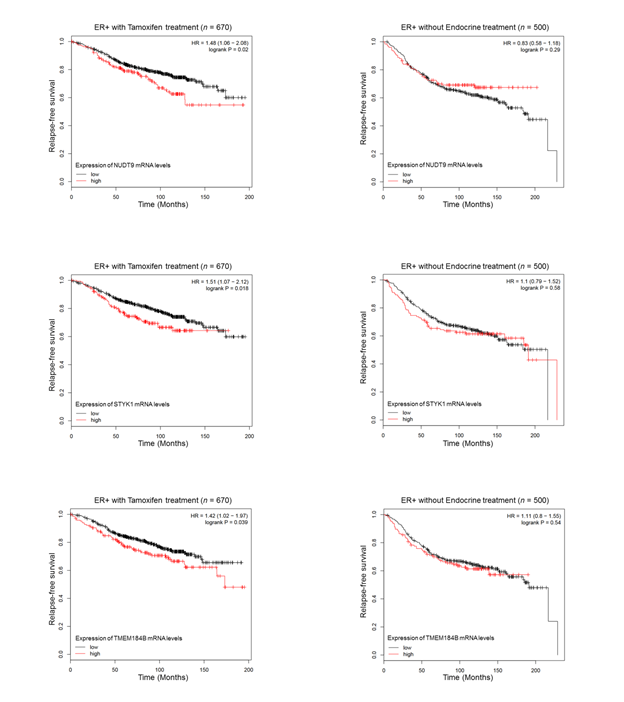


**Figure S15.** Relapse-free survival analysis of NUDT9, STYK1 and TMEM184B in ER+ patients. The *p* value was computed with the log-rank test. Analysis was referred to the published paper (Lanczky et al. 2016). HR: Hazard Ratio.


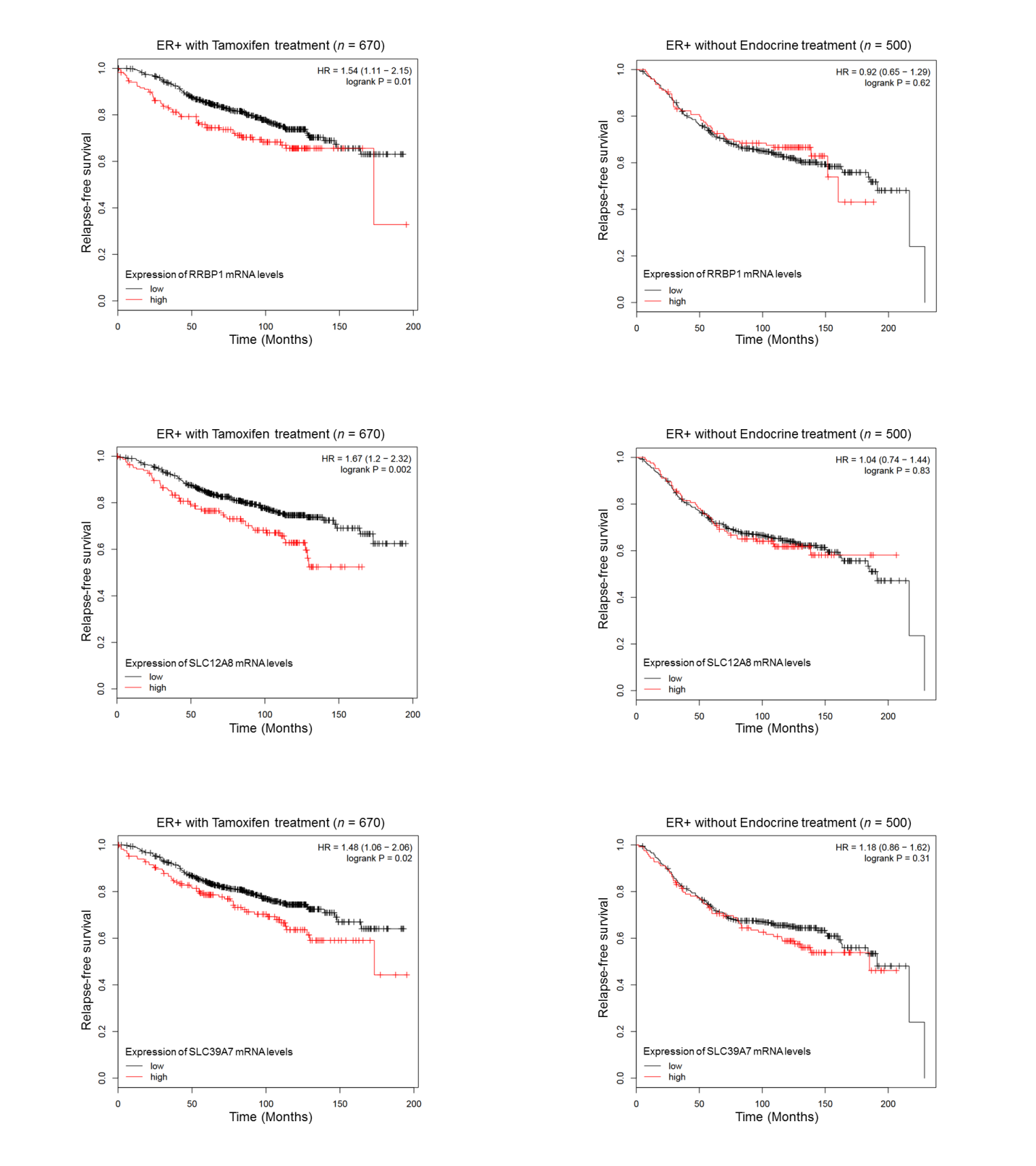


**Figure S16.** Relapse-free survival analysis of RRBP1, SLC12A8, SLC39A7 in ER+ patients. The *p* value was computed with the log-rank test. Analysis was referred to the published paper (Lanczky et al. 2016). HR: Hazard Ratio.


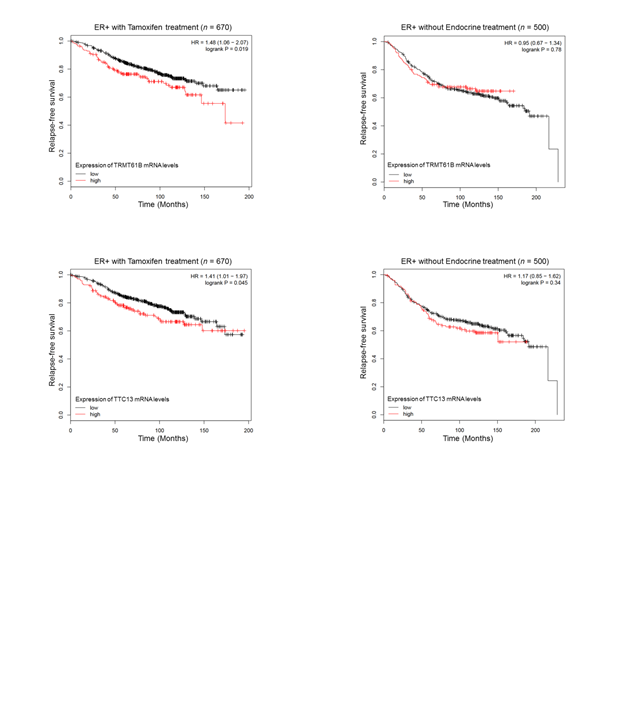


**Figure S17.** Relapse-free survival analysis of TRMT61B, TTC13 in ER+ patients. The *p* value was computed with the log-rank test. Analysis was referred to the published paper (Lanczky et al. 2016). HR: Hazard Ratio.

**Supplementary Tables**

**Table S1.** Public Hi-C datasets used in HiSIF training.

| **Data set** | **Enzyme** | **Read length** | **Sequencing depth**  **(PE reads)** | **RSS (kb)** | **Time cost*** |
| --- | --- | --- | --- | --- | --- |
| GM12878 in situ Hi-C | MboI | 101 | 5,594,884,851 | 244,612,928 | 3:11:43 |
| hESC Hi-C | HindIII | 50 | 1,999,419,070 | 62,174,832 | 37:40 |
| MCF7 Hi-C | HindIII | 101 | 286,286,296 | 110,767,424 | 12:28 |
| MCF10a Hi-C | HindIII | 101 | 289,975,071 | 113,026,736 | 10:27 |
| K562 | MboI | 101 | 1,366,228,845 | - | - |

*Time cost was calculated by running HiSIF on public dataset using the configuration: AMD 2.4GHz, 64 Cores, 256Gb Memory.

**Table S2.** Primers for 3C-qPCR.

| Name | Primer |
| --- | --- |
| GAPDH_Anchor | ATGCAAGGCTTTCTCTTAAATTAGC |
| GAPDH_Control | AATTCTGAGCATTCTGTAGCAAACT |
| ACP1_Anchor | CCTTATATTATTTTCTTCAATGGAC |
| ACP1_24K | AGAGATCTTGGGCAGACAGAGAGAC |
| ACP1_31K | ATCCATATCCAATTTTTATGATTTA |
| ACP1_42K | CAGACAACTATGGGATGGGTAAGAG |
| ACP1_52K | AAGTGGGTATTGACTTTAACAAAAT |
| ACP1_61K | GTCAGAGTTCTGAAATGGTTGTTTT |
| ACP1_70K | GTGTTGCTACATAATCAACATTTTA |
| ACP1_79K | ATTGCCTTTCTTATTGATATAGGAG |
| ACP1_89K | GGAAGGCTTACTTTGTAAGATGTTT |
| ACP1_99K | TTAAACAATTAATTCAAAGCAACTA |
| HECTD1_Anchor | TTATTAGGTGATATTGTCAGGTACA |
| HECTD1_21K | CTTTGTAACCAAATAAAAATGAAAT |
| HECTD1_31K | GTCTATAACCAAGTTTTCTCTGGTC |
| HECTD1_40K | ATAGGTAACAGATGAGGAAGAAATC |
| HECTD1_50K | CAGGAGGTCACTCTACTTTACACAT |
| HECTD1_61K | TTGTTTGTTTTAACACTTCGTTACT |
| HECTD1_68K | TAAAAAGGTGAAAAAGTTTTGTCTG |
| HECTD1_82K | AGGTGTTATATCCAGTGTTATTTTA |
| HECTD1_90K | ATTATCTCTCATTTTATGGACAAGA |
| MBIP_Anchor | TTTTGTAATCTTTTAGTCAGACTCC |
| MBIP_21K | TACTGCAGCAAAGAAAAAGTAAAAT |
| MBIP_30K | ATAACATGGCAGTAAATAGGTTTAT |
| MBIP_41K | TTTAGGAACAAATACTTCCCTTTTA |
| MBIP_51K | CACACTATAATACAGAAAAGTAAAG |
| MBIP_60K | TAGCCGAATAATTTATGACTAAGAC |
| MBIP_68K | GCTAGAAACTAGCATTATCTCATTA |
| MBIP_78K | CAGAGGTAGTTAAGCAAAGAATCTG |
| MBIP_89K | GTAGGGGCCAACAGACACCTCATAC |
| MBIP_99K | ACTGACTTAGATGGTGAAGCTGAAA |

**Table S3.** Primers for RT-qPCR.

| Name | Primer |
| --- | --- |
| GAPDH_F | ATGTTCGTCATGGGTGTGAA |
| GAPDH_R | TGTGGTCATGAGTCCTTCCA |
| ACP1_F | GTTTTCAGGAAACTTGTAAC |
| ACP1_R | CTATCTCATACCCGGAAGTT |
| HECTD1_F | GATGATAATTTTCCAGATGA |
| HECTD1_R | CTTTCAAGCTCATCACTATC |
| MBIP_F | AATTGACAGACGAATATCTG |
| MBIP_R | GTTCTTGCACAACTATTTTC |

**References:**

Akdemir KC, Chin L. HiCPlotter integrates genomic data with interaction matrices. Genome Biol. 2015;Sep 21;16:198. doi: 10.1186/s13059-015-0767-1.

Crane E, Bian Q, McCord RP, Lajoie BR, Wheeler BS, Ralston EJ, Uzawa S, Dekker J, Meyer BJ. Condensin-driven remodelling of X chromosome topology during dosage compensation. Nature. 2015;Jul 9;523(7559):240-4.

Dixon JR, Jung I, Selvaraj S, Shen Y, Antosiewicz-Bourget JE, Lee AY, Ye Z, Kim A, Rajagopal N, Xie W, Diao Y, Liang J, Zhao H, Lobanenkov VV, Ecker JR, Thomson JA, Ren B. Chromatin architecture reorganization during stem cell differentiation. Nature. 2015;518(7539),331-6.

Lanczky A, Nagy A, Bottai G, Munkacsy G, Paladini L, Szabo A, Santarpia L, Gyorffy B. miRpower: a web-tool to validate survival-associated miRNAs utilizing expression data from 2,178 breast cancer patients. Breast Cancer Res Treat. 2016;160(3):439-446.

Rao SS, Huntley MH, Durand NC, Stamenova EK, Bochkov ID, Robinson JT, Sanborn AL, Machol I, Omer AD, Lander ES, Aiden EL. (2014) A 3D map of the human genome at kilobase resolution reveals principles of chromatin looping. Cell. Dec 18;159(7):1665-80.
